# Extended Reality Biofeedback for Functional Upper Limb Weakness: Mixed Methods Usability Evaluation

**DOI:** 10.2196/68580

**Published:** 2025-06-03

**Authors:** Anirban Dutta, Katerina Hatjipanagioti, Matthew Alexander Newsham, Lewis Leyland, Lindsey Rickson, Alastair Buchanan, Ildar Farkhatdinov, Jacqueline Twamley, Abhijit Das

**Affiliations:** 1 Department of Metabolism and Systems Science, University of Birmingham, Edgbaston, Birmingham, B15 2TT, United Kingdom, 44 7395260645; 2 FND North, Heysham Morecambe, United Kingdom; 3 Royal Preston Hospital, Preston, United Kingdom; 4 Nudge Reality Ltd, Cheshire, United Kingdom; 5 School of Biomedical Engineering and Imaging Sciences, King's College London, London, United Kingdom; 6 Lancashire Teaching Hospitals NHS Foundation Trust, Preston, United Kingdom; 7 University of Central Lancashire, Preston, United Kingdom

**Keywords:** extended reality, haptics, functional neurological disorder, biofeedback, usability, co-design, System Usability Scale

## Abstract

**Background:**

The perception–action cycle enables humans to adapt their behaviors by integrating sensory feedback into motor actions. Functional neurological disorder (FND) disrupts this cycle, leading to maladaptive motor responses and a diminished sense of agency. FND includes functional seizures, movement disorders, and cognitive impairments, significantly affecting quality of life. Recent advancements in extended reality (XR) neurotechnologies provide opportunities for novel rehabilitation approaches, leveraging visual and haptic feedback to retrain motor control and restore agency in individuals with functional limb weakness.

**Objective:**

This study aimed to co-design and evaluate an XR-based biofeedback platform for upper-limb rehabilitation in FND, incorporating multisensory feedback (visual and haptic) to enhance motor retraining.

**Methods:**

A mixed methods design was used. In phase 1, a Delphi survey (N=20, patients with FND) identified key user requirements, emphasizing customizability, real-time feedback, accessibility, and comfort. These insights guided the codevelopment of an XR biofeedback platform. In phase 2, a co-design workshop with 6 participants (3 FND patient representatives and 3 health care professionals) evaluated the usability of 3 XR training tasks: virtual reality (VR) relaxation task, a guided meditation in a VR calming environment; XR position feedback task (“Hoop Hustle”), a VR-based motion task requiring arm movements to interact with virtual objects, providing real-time positional biofeedback; and XR force feedback task, a haptic robot-assisted exercise using the Human Robotix System (HRX-1) haptic device, applying resistive forces to guide upper limb movements. Participants completed system usability scale (SUS) questionnaires and provided qualitative feedback, which was analyzed using NVivo (QSR International) thematic analysis.

**Results:**

The XR position feedback task achieved the highest usability ratings, with 4 out of 6 participants scoring it above 85, indicating “excellent” usability. The VR relaxation task received polarized scores: 2 participants rated it highly (90 and 87.5), while 3 scored it poorly (mid-40s), citing motion discomfort and disengagement. The XR force feedback task had mixed usability outcomes (SUS range: 27.5‐95.0), with 1 participant with functional dystonia struggling significantly (SUS 27.5), while others rated it between 62.5 and 95.0. Qualitative feedback emphasized comfort (lighter headsets and better ergonomic design), immersion and content quality (clearer visuals and reduced distracting audio prompts), personalization (adjustable settings for speed, difficulty, and force resistance), and accessibility (cost concerns and home usability considerations). Overall, participants viewed the XR biofeedback platform as highly promising but in need of fine-tuning.

**Conclusions:**

This study demonstrates the feasibility and usability of an XR neurotechnology platform for FND rehabilitation, with strong acceptance of XR position feedback, mixed reactions to VR relaxation, and individual-specific usability outcomes for the force feedback task. Findings underscore the need for personalization features and hardware refinement. Future work will focus on enhancing usability, improving accessibility, and evaluating effectiveness in larger clinical trials.

## Introduction

Humans continuously learn through interactions with their environment via a perception-action cycle—a feedback loop where sensory input informs actions and the consequences of these actions (shaped by rewards and penalties) reinforce or modify behavior over time. This adaptive learning process is crucial for navigating social and environmental contexts, allowing individuals to align their behaviors with societal norms and expectations. However, maladaptive learning can occur when responses to rewards and penalties lead to dysfunctional behavior patterns, diminishing an individual’s sense of agency and resulting in disordered actions [1]. We hypothesize that functional neurological disorder (FND) may arise from such maladaptive learning within the perception-action cycle, where certain reinforced behaviors disrupt normal functional responses, contributing to symptoms and reduced voluntary control over bodily actions.

FND is a complex, debilitating condition with symptoms comparable in severity and societal cost to those of epilepsy or multiple sclerosis [2]. FND encompasses several subtypes—functional seizures, functional movement disorders, persistent perceptual postural dizziness, and functional cognitive disorder—stemming from interplay between neurological and psychological mechanisms [3]. Yet, only about 50% of United Kingdom health boards have established care pathways for FND, underscoring significant gaps in treatment [4]. Recent advancements in neurotechnology and better understanding of FND pathophysiology have revealed shared mechanisms (such as abnormal sensorimotor processing and disruptions in sense of agency) that can be targeted by novel therapeutic strategies [3]. Notably, extended reality (XR) approaches have been proposed within a stepped-care rehabilitation framework [5], enabling interventions to be tailored based on symptom severity and delivered from clinic to home settings. XR is an umbrella term encompassing immersive technologies that blend digital and physical environments, including augmented reality (AR), virtual reality (VR), and mixed reality (MR). AR overlays digital information onto the real world, VR fully immerses users in a computer-generated environment, and MR allows interactive overlay of artificial elements onto the real world. XR platforms can incorporate haptic (touch-based) feedback and guided suggestions to engage patients through bottom-up sensory input and top-down cognitive cues, respectively, aiming to retrain the disrupted perception-action links underlying FND symptoms [6]. For example, haptic feedback may provide real-time physical cues to encourage movement, while positive verbal reinforcement (“You’re doing great!”) can facilitate operant conditioning during VR rehabilitation [7].

A survey of 527 individuals revealed high comorbidity rates among patients with FND, with pain (78.1%), fatigue (78.0%), and sleep disturbances (46.7%) being the most common symptoms, often worsening postdiagnosis [8]. Effective FND management underscores the need for transparent diagnosis explanations to improve patient understanding and enable personalized treatment strategies [9]. The National Institute of Mental Health’s Research Domain Criteria framework [10] offers a dimensional perspective for understanding FND [11], guiding the development of neurotechnologies and biomarkers to better categorize its heterogeneity. The recent proposal for the inclusion of the sensorimotor domain in the Research Domain Criteria highlights the growing recognition of sensorimotor processing in mental health [10], presenting opportunities for intervention through XR neurotechnologies.

Building on previous VR-based interventions [12], we proposed the integration of haptic feedback into an XR setting to modulate the balance between sensory attenuation and amplification using an operant conditioning framework [7]. Haptic feedback in visuo-motor tasks plays a crucial role in reinforcing the perception-action cycle, primarily through efference copy and corollary discharge integration, which differs from motor imagery-based VR training [13]. The efference copy is an internal duplicate of motor commands from the supplementary motor complex [14], allowing the cerebellum and sensory areas to predict sensory consequences of movement [15]. This predictive function enables the brain to distinguish between self-generated actions and external stimuli, an essential aspect of sensorimotor learning. When haptic feedback is absent, motor learning relies on mental simulations without new sensory data, potentially reinforcing maladaptive internal models, as observed in cerebellar dysfunction [13,16]. In adaptive XR learning, haptic feedback serves as real-world sensory input, aiding in the recalibration of maladaptive internal models and reducing overreliance on predictive mechanisms associated with mental simulations in VR-only settings. Studies show that without haptic input, individuals struggle to correct motor prediction errors, as their internal model fails to recalibrate effectively [17]. By integrating haptic feedback into XR rehabilitation, we aim to recalibrate maladaptive sensorimotor patterns related to fatigue (effort-reward mismatch [18]), pain, weakness, dystonia, and seizures.

**Figure 1.**
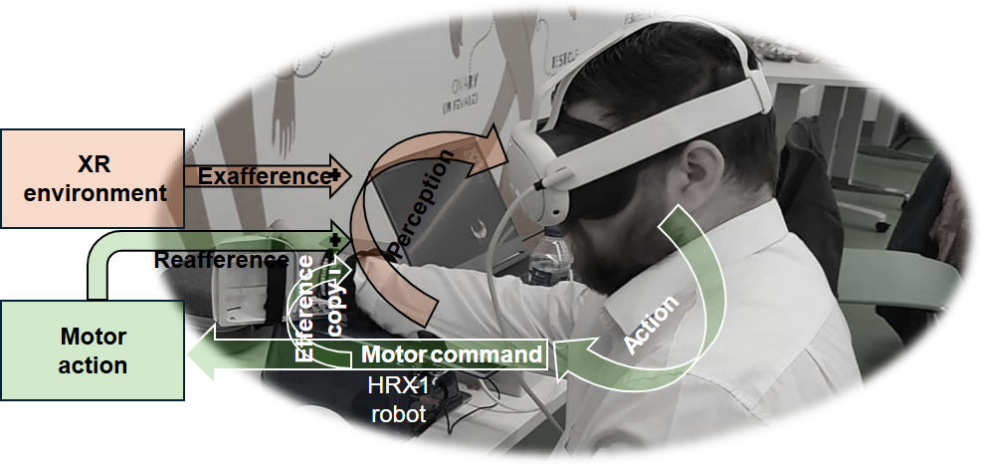
Perception-action coupling for extended reality (XR) biofeedback training to modulate bottom-up reafference with exafference through a haptic robot (HRX-1) to support movement in cases of functional weakness. Top-down modulation is influenced by guided visual and verbal suggestions presented via XR feedback. A distinction can be made between efference copy—internal brain duplicates of motor commands (in action)—and corollary discharge, which involves expected sensory signals due to those motor commands (in perception).

Support for XR-based functional motor disorder (FMD) rehabilitation also stems from intentional binding research, which suggests that repeated operant experiences enhance implicit agency by reinforcing associative learning [19]. This highlights the distinction between explicit and implicit agency: explicit agency, tied to conscious awareness, can be strengthened through demonstrations like Hoover’s sign or tremor entrainment [20], while implicit agency is shaped through repeated operant conditioning [7]. These mechanisms interact via top-down and bottom-up pathways, which can be experimentally modulated in XR through exafference—the controlled simulation of external stimuli. However, the ethical, cost, and usability concerns associated with digital health interventions necessitate stakeholder engagement to ensure alignment with broader health care goals. Industry-driven digital health innovation plays a key role in assessing how these technologies impact health care systems and patient outcomes. Our research focuses on evaluating the usability of an XR neurotechnology platform for biofeedback training in functional limb weakness, combining bottom-up haptic feedback with top-down visuo-motor task suggestions (refer to Figure 1) [6]. The ultimate goal is to develop precise, technically effective, sustainable, and patient-friendly XR neurotechnologies for FND rehabilitation. Industry-driven innovation plays a key role in translating these technologies into practice by evaluating their impact on health care systems and outcomes. The ultimate objective is to ensure that such neurotechnology is not only effective but also user-friendly, acceptable, and accessible for people with FND. In this context, this study adopted a coproduction approach to co-design an XR biofeedback training platform for functional limb weakness in FND and to assess its usability with end-users.

## Methods

### Study Setting and Participants

This study consisted of two phases: an exploratory survey (Delphi method) conducted online to inform platform design and a subsequent in-person co-design workshop for usability evaluation.

In phase 1, an exploratory Delphi survey was conducted online, where a convenience sampling method was used to recruit individuals with lived experience of FND as “experts by experience” from the United Kingdom Royal Preston Hospital’s FND service team’s networks led by the PPIE (Patient and Public Involvement and Engagement) leads. In total, 20 individuals (experts by experience) with FND participated in the initial round of the Delphi survey. Participants provided feedback via an online questionnaire. The survey collected both quantitative and qualitative data on several topics: familiarity with VR and haptic technologies, perceptions of comfort and ease of use, anticipated relevance and impact of an XR-based therapy for FND, and potential barriers to adoption (such as, cost, access to equipment, technical support, and side effects). Responses were analyzed to extract common themes and requirements that the PPIE lead presented at the National Rehabilitation Centre (NRC) Rehabilitation Technologies Conference 2024 [21] (NRC Rehabilitation Technologies Conference 2024 poster and slides in Multimedia Appendix 1). Based on the survey findings, we codeveloped with the PPIE leads and industry partners (Human Robotix Ltd and Nudge Reality Ltd) a prototype XR neurotechnology platform. We selected the Human Robotix HRX-1 upper-limb haptic system (a portable robotic device providing force feedback) and Nudge Reality’s “Hoop Hustle” XR game as the core components for our platform, as these were judged by the PPIE leads to best meet the identified needs (detailed specifications of the hardware and game options are available in Multimedia Appendix 2). The HRX-1 device can assist or resist arm movements with precise torque control, while Hoop Hustle is a VR game that can be adapted for therapeutic exercises.

For the phase 2 co-design and usability testing workshop, a purposive sampling approach was used to recruit participants specifically from the United Kingdom Royal Preston Hospital’s FND Service team, including PPIE leads. We then conducted an in-person workshop involving 6 participants drawn from the FND service community: 3 FND patient representatives (1 female and 2 male) and 3 health care professionals (2 physiotherapists and 1 neurologist; 2 female and 1 male). All 6 participants are coauthors of this paper for the participatory design approach. Before the workshop, participants provided informed consent. The session took place in a rehabilitation clinic setting and lasted about half a day.

### Ethical Considerations

As this work was part of a patient engagement and technology co-design project, it was conducted with institutional review board notification but was determined to be a service development and quality improvement activity not requiring full National Health Service (NHS) Research Ethics Committee review. All participants gave written informed consent for their involvement and for publication of deidentified feedback. The study was carried out in accordance with the Declaration of Helsinki principles of ethical research.

### Procedure and XR Platform Tasks

Given the selection of Human Robotix’s HRX-1 system for upper limb rehabilitation (Human Robotix’s HRX-1 system in Multimedia Appendix 3) and Nudge Reality’s “Hoop Hustle” game (Nudge Reality’s XR games in Multimedia Appendix 4) by PPIE leads, efforts were focused on adapting these technologies to test 3 conditions: VR relaxation, XR positional feedback, and XR force feedback. During the co-design workshop, the prototype XR platform was introduced, and participants were guided through 3 interactive training tasks, each representing a different mode of biofeedback.

#### Experimental Robotic System

**Figure 2.**
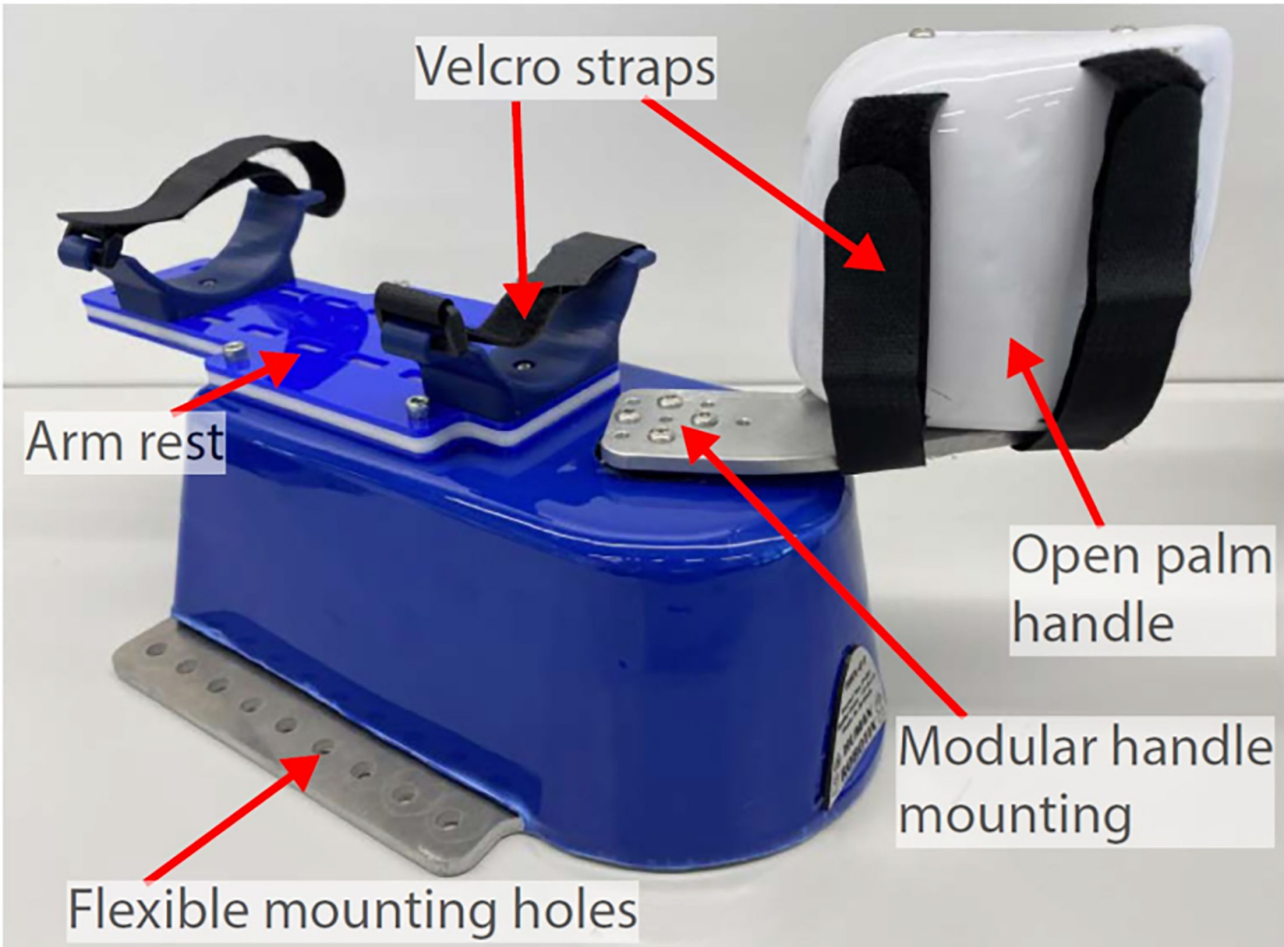
HRX-1 robot that can generate programmable wrist flexion and extension torques for assistance or resistance during the experimental study.

A 1-degree-of-freedom HRX-1 desktop robot (refer to Figure 2) equipped with a direct-drive electromagnetic motor for wrist flexion or extension movement was used in the study. The robot offers high flexion or extension torque (up to 2 Nm), position and torque sensing, and a variety of control modes in a compact robotic platform. The design of the HRX-1 robot is substantially more compact and lighter than existing comparable systems to enable easy transportation and installation for the studies in clinical, research, and at-home environments. The safety of the robot operation was implemented at mechanical (range of motion limitation with end-stops), electric (limitation for the maximal electric current), and software (limitation on the maximal speed of movement) levels. Previously, robots have been successfully used in clinical and research studies [22-24]. In this study, the HRX-1 robot was integrated with VR tasks.

#### VR Relaxation Task

Participants wore a Meta Quest 3 VR headset to experience a guided relaxation session. The VR environment featured calming scenery (eg, a gradually descending landscape or serene nature scene), accompanied by a gentle narrative instructing the user in relaxation techniques (for instance, breathing exercises, and progressive muscle relaxation cues). The purpose of this task was to familiarize users with VR and induce relaxation, which can help reduce FND symptom intensity. Participants remained seated during this task. Notably, based on user feedback from the Delphi survey, we avoided any instructions that would conflict with VR immersion (as one Delphi respondent cautioned that this could cause disorientation). The task lasted about 5‐7 minutes.

#### XR Position Feedback Game (Hoop Hustle)

In this task, participants engaged with hoop hustle, a therapeutic game developed for XR rehabilitation. The user’s goal in the game is to move their affected arm (or a controller held in that arm) to “shoot” balls in VR through a series of hoops or targets at varying positions. The game provides real-time visual feedback on the accuracy and speed of the user’s arm movements. For example, when a participant moves their arm, a corresponding arm or cursor in VR is shown, allowing them to adjust their movement to align with the hoop. Successful hits (getting the ball through the hoop) trigger immediate positive feedback (visual effects and encouraging sounds). The game’s difficulty can be adjusted—for example, hoop height and size can be modified to accommodate the user’s range of motion, and the speed of ball generation can be tuned. During the workshop, an operator adjusted these settings as needed to ensure each participant could comfortably attempt the task. This task emphasized positional biofeedback (augmented visual feedback of movement) without additional force resistance. Each participant practiced for several minutes until they felt they had experienced the core mechanics of the game.

#### XR Force Feedback Task

The HRX-1 haptic robot was integrated with the hoop hustle game to provide force feedback during the exercise. Participants grasped the end-effector of the HRX-1 device, which was programmed to apply gentle resistive forces or assistance during specific arm movements in the VR game. For instance, as a participant guided a ball toward a hoop in VR, the device might add a slight downward resistance, requiring the user to exert additional effort and thus engage proprioceptive feedback pathways. In this way, the XR force feedback task combined visual and haptic biofeedback. We also implemented a simple exercise game: the wrist handle of the robot was used to control a visual cursor shown in the screen, and a participant’s task was to rotate the handle with their wrist follow a pseudo-random movement of a target on the screen as accurate and as fast as possible, similar to the tasks used in [25]. A participant could observe the progress task on the screen (visual modality) and feel the assistive and resistive wrist flexion or extension torques generated by the robot (force feedback modality). This was included to explore how force feedback might help reveal or train aspects of motor control in FND (eg, addressing sensory attenuation deficits). Each participant spent around 5 minutes with force feedback enabled. One participant with functional dystonia required a brief rest during this task due to muscle fatigue; however, all participants were able to attempt the task to some extent.

Throughout the session, participants were encouraged to “think aloud” and share any difficulties or observations (eg, if the headset felt uncomfortable or if a task was confusing). A facilitator took notes on these observations to supplement the formal feedback.

### Data Collection and Analysis

After completing all 3 tasks, participants filled out the system usability scale (SUS) questionnaire for each task. The SUS is a 10-item questionnaire yielding a score from 0 to 100, where higher scores indicate better perceived usability. We chose the SUS because it is a well-established, quick tool for usability assessment, suitable even for small samples [26]. Participants also provided written free-text feedback on their experience with each task and the overall platform. These responses were collected on paper forms and later transcribed. In addition, the workshop concluded with a short group discussion, allowing participants to collectively reflect on what aspects of the platform worked well and what improvements they would prioritize. The discussion was later summarized in notes.

Quantitative data from the SUS were summarized using descriptive statistics, given the small sample size. We report individual SUS scores per participant and per task, as well as the range and median for each task’s scores. Following convention [27], we interpret SUS scores using an adjective rating scale for context: scores above~85 are considered “excellent,” around 70‐85 “good,”~50‐69 “okay,” and below 50 “poor” in terms of usability perception. We did not perform inferential statistical tests due to the exploratory nature of this pilot and the limited number of subjects. Qualitative data (written feedback and facilitator notes) were analyzed thematically. Two researchers (1 patient representative and 1 study investigator) independently reviewed the feedback to identify recurring themes. Using NVivo 12 (QSR International), feedback comments were coded with initial labels corresponding to aspects of user experience (eg, “hardware discomfort,” “audio feedback,” and “game difficulty”). These codes were then grouped into higher-level themes through discussion and consensus. Representative participant quotes were extracted to illustrate each theme in the Results.

## Results

### Phase 1: Exploratory Delphi Survey Report (CHERRIES Checklist)

We present the results from our first round of the Delphi survey according to the Checklist for Reporting Results of Internet E-Surveys (CHERRIES) [28], aimed at the translation of the VR haptics technology for biofeedback training in FND. The survey gathered online feedback from 20 (N=20) individuals with lived experience of FND, considered experts by experience for technology translation.

#### Design

This was an online Delphi survey aimed at gathering high-level user requirements for the development of a VR haptics biofeedback training platform for FND rehabilitation.

The survey sought to assess perceptions and expectations of VR and haptic biofeedback technology for rehabilitation, potential benefits and usability considerations for upper and lower limb motor retraining, and barriers to adoption and accessibility concerns among individuals with lived experience of FND.

#### Development and Pretesting

##### Survey Development

The survey was co-designed by a multidisciplinary team (co-authors of this report), including clinicians, researchers, industry partners, and FND patient representatives. It was pilot-tested with a small group of patients with FND and clinicians to refine clarity, content, and usability.

##### Survey Refinements

Feedback from pilot testing led to revisions in question phrasing, response categories, and survey logic. Adjustments were made to ensure accessibility for individuals with neurological impairments (eg, clear navigation and avoiding long response formats).

### Recruitment Process and Sample Characteristics

#### Target Population

The survey targeted adults (≥18 y) with FND, particularly those experiencing functional limb weakness.

#### Recruitment Strategy

Participants were recruited through FND patient advocacy organizations (eg, FND Hope, FND Action). Neurology clinics specializing in FND care. Online FND support groups and social media communities. The survey link was shared via email, social media, and organizational websites.

#### Participation Details

Participation details included a survey link access, in which an open-access URL was provided with IP duplicate detection enabled. No monetary incentives were provided; participants were thanked for their contributions in follow-up communications.

### Survey Administration

The survey was hosted on a General Data Protection Regulation (GDPR)-compliant, secure online platform (MS Forms is part of Microsoft 365, which adheres to GDPR, Health Insurance Portability and Accountability Act (HIPAA), and ISO 27001 security standards).

### Response Tracking

Anonymous participation was allowed; no email registration was required, and no IP tracking or cookies were used.

### Survey Content

The question structure of the survey included a combination of question types, as listed in Textbox 1.

Textbox 1. **Question structure of the survey.**

- Demographics (age, gender, FND diagnosis history, and previous XR or VR experience).
- Experience with VR or haptic technology (previous use in gaming, therapy, etc).
- Perceived benefits of XR biofeedback (customizability, real-time feedback, and usability).
- Barriers to adoption (cost, accessibility, and concerns about motion sickness).
- Open-ended qualitative feedback (expectations, concerns, and usability considerations).

### Data Handling and Statistical Analysis

#### Data Privacy Measures

No personally identifiable information was collected. Responses were stored in a secure, encrypted database, accessible only to authorized researchers.

#### Analysis Methods

Descriptive statistics were used for Likert-scale responses (percentages and means). Qualitative thematic analysis was performed using NVivo for open-ended responses.

### Results Reporting

#### Response Rate

Response rates are described in Textbox 2.

Textbox 2. **Response rate.**

- Total respondents: 20.
- Completion rate: 85% (17 fully completed responses).
- Dropout rate: 15% (3 partial responses).

#### Key Findings

Key findings are mentioned in Textbox 3.

Textbox 3. **Key findings.**

- Participant demographics:
  - Peak age group: 35-44 years.
- Gender: predominantly female.
- Experience and perception of VR and haptic technology
  - Awareness of VR technology: high, but varied levels of familiarity.
  - Haptic technology experience: less common.
- Comfort levels: mostly positive, but some concerns about mask and goggle discomfort and motion sickness.
- Perceived relevance and potential impact: high perceived relevance for FND rehabilitation.
- Participants prioritized:
  - Customizable exercises.
  - Real-time biofeedback.
  - Immersive environments.

- Barriers and challenges identified
  - Accessibility concerns: (1) cost of VR equipment, (2) availability through NHS or insurance coverage, (3) WiFi or connectivity limitations.

- Usability issues:
  - Motion sickness concerns.
  - Need for guidance on using XR biofeedback at home.

- Potential safety concerns:
  - Risk of falls or overstimulation.

### Discussion of Bias and Limitations

#### Potential Biases

The two types of potential biases are (1) selection bias: participants were self-selected, possibly favoring tech-savvy individuals, or those already engaged in FND support groups; and (2) response bias: some participants may have been overly optimistic or cautious in their feedback.

### Limitations

This study has two limitations. The first is the small sample size (N=20); the results are preliminary and not generalizable to all patients with FND. The second is the use of the single-round Delphi survey; the findings require further validation through additional rounds or larger-scale studies.

### Conclusion

The first round of the Delphi survey provided key insights into the usability, expectations, and barriers associated with XR haptics biofeedback training for FND rehabilitation.

### Key Takeaways

Participants perceived high potential benefits but highlighted cost, accessibility, and usability concerns. There was a strong interest in real-time feedback and customization to tailor the technology to individual needs. Concerns about motion sickness, equipment comfort, and NHS availability need to be addressed for successful adoption.

### Future Steps

Refining usability features based on patient feedback in phase 2 co-design and usability testing. Further stakeholder engagement with clinicians, patient organizations, and industry partners in phase 2 co-design and usability testing. Scaling the study to validate findings with a larger sample and additional Delphi rounds following in phase 2 co-design and usability testing.

### Phase 2: Usability Scores (Quantitative Results)

Basic usability testing typically benefits from the purposive selection of 5‐10 participants [29]. Here, all 6 workshop participants completed the XR position feedback and XR force feedback tasks, and 5 completed the VR relaxation task (1 health care professional was unable to try the VR relaxation due to time constraints). Table 1 presents the SUS scores given by each participant for each task. Overall, the XR position feedback game received the highest ratings with a median score of 91.3, and all participants rated it above 70. The VR relaxation task had a bimodal distribution of scores—2 participants rated it very highly (~88‐90) while 3 participants gave it scores below 50, indicating poor usability for those individuals. The XR force feedback task had generally positive scores from 4 participants (range, 80.0‐95.0), but 1 participant (Participant 3) gave a very low score (27.5). According to Bangor et al’s [27] adjective rating scale for SUS, the low scores in the 40s for the VR relaxation task correspond to a “poor” usability experience, despite the same task being rated as “excellent” by others. Similarly, the force feedback task’s scores suggest mostly “good” to “excellent” usability, with one clear outlier in the “poor” range. In contrast, the XR position feedback task’s scores correspond to “good” or “excellent” usability across all users. These results highlight a high degree of variability in user experience for the more complex or condition-sensitive tasks (VR relaxation and force feedback), compared to the consistently positive experience with the position feedback game (XR system usability testing script and XR system usability testing results in MultimediaAppendices 5^8^).

**Table 1.** The system usability scale (SUS) score was calculated for each participant across extended reality (XR) tasks, including virtual reality (VR) relaxation, XR position feedback control, and XR force feedback control. Participant 6 did not participate in the VR relaxation task.

	XR force feedback SUS	XR position feedback SUS	VR relaxation SUS
Participant 1	80.0	95.0	47.5
Participant 2	62.5	72.5	90.0
Participant 3	27.5	92.5	45.0
Participant 4	82.5	100.0	87.5
Participant 5	95.0	90.0	45.0
Participant 6	87.5	85.0	—^a^

a not available.

### Phase 2: User Feedback and Thematic Analysis (Qualitative Results)

Qualitative analysis of the feedback revealed several key themes regarding the user experience and suggestions for improvement. Participants provided free-text responses regarding their VR relaxation task experience, which were analyzed for future technology improvement.

#### Immersion and Visual Artifacts (Improve Realism and Reduce Pixelation)

Some participants struggled with visual quality, stating that the graphics were “bland” and “pixelated.” One participant mentioned, “The environment didn’t feel real enough to help me relax.”

#### Discomfort With the Headset (Select Lighter Weight Hardware)

Participants found the VR headset too heavy, making it difficult to use for prolonged relaxation. One user commented, “The headset was too bulky—it distracted me rather than helping me relax.”

#### Voice Guidance Issues (Offer Customizable Audio Settings)

While some users appreciated the guided relaxation, others found the voiceover distracting or repetitive. One participant stated, “The voice instructions were too constant—I wanted more silence to focus on breathing.”

#### Motion Sickness and Unpleasant Sensations (XR May Minimize Some Disorienting Effects)

A few participants experienced dizziness, with one stating, “The moving visuals made me feel nauseous, which completely defeated the point of relaxing.” This suggests a need for less intense motion effects.

#### Mixed User Feedback on Effectiveness (Offer Alternatives, Eg, Audio-Only Modes)

Some participants felt the VR relaxation could be beneficial if improved, while others stated they would prefer alternative relaxation methods (eg, audio-only relaxation without VR).

Participants also provided free-text responses regarding their experience with the XR position feedback task, which were analyzed for future technology improvement.

#### Real-Time Visual Feedback Issues (Lower Latency Motion Tracking)

Some participants struggled with feedback clarity, reporting inconsistencies in motion tracking. One participant noted, “Sometimes my arm was perfectly aligned, but it wouldn’t register the movement.”

#### Difficulty in Adjusting Position (Online Recalibration)

A few participants found it difficult to match their movements with the system’s feedback. One participant commented, “I kept missing the hoop even when I thought I was on target.” Another commented, “I liked that it gave immediate feedback, but sometimes I didn’t understand what I did wrong.” This suggests that target alignment and hit detection need refinement.

#### Engagement and Gamification Elements (Expand Game-Like Elements)

Some participants enjoyed the interactive aspect of the task. One participant stated, “It was fun trying to score points, but I wish there were more levels or challenges.”

#### Physical Strain Concerns (Individualized Task Intensity)

A small number of participants reported discomfort or strain during prolonged use. One participant mentioned, “I could feel my arm getting tired quickly—I think the tracking required more effort than I expected.”

#### Mixed User Feedback on Usability (Lower Latency Motion Tracking and Online Recalibration)

Some participants felt that improving the accuracy and responsiveness of the tracking would make the task more engaging. One participant suggested, “If it was more precise in detecting movements, I’d find it much more enjoyable.”

Participants provided additional free-text comments about their experience using the XR force feedback task, which were analyzed for future technology improvement.

#### Lack of Personalization (Individualized and Adaptive Resistance)

Several participants noted that the resistance levels were not well-adjusted to their needs. One participant stated, “The force applied felt either too weak or too strong—there was no in-between.” This suggests a need for adaptive resistance control.

#### Discomfort and Fatigue (Improve Ergonomics)

The heaviness of the headset and the effort required to overcome force resistance were cited as major concerns. One participant reported, “After a few minutes, my arm felt very fatigued, which made the task frustrating rather than helpful.” Another stated, “The device felt restrictive rather than supportive.”

#### Low Engagement (Expand Game-Like Elements)

The lack of an interactive or gamified element was also highlighted. One participant commented, “There’s no motivation to keep going—it’s just moving against resistance with no real feedback.”

#### Potential for Improvement (Future Potential)

Some participants saw promise in the concept but suggested improvements, such as, “It would help if the system guided me on whether I was applying the right force,” “Maybe add vibration or a sound effect when I get the force correct,” and “If the resistance could change based on how strong I am, that would be much better.”

#### Summary

In summary, the qualitative feedback provided actionable information that complemented the SUS usability scores. It explains why certain tasks received lower scores (eg, VR relaxation’s technical and content issues leading to poor ratings from half the group) and reinforces the need for customization in the force feedback task (given one user’s difficulties). The participatory nature of the co-design and usability testing session ensured that end-user voices directly informed the next steps of platform refinement.

## Discussion

### Principal Results

This study is, to our knowledge, the first mixed methods evaluation of an XR-based biofeedback training platform co-designed for individuals with motor FND. Through a 2-phase coproduction approach, we obtained rich stakeholder input and preliminary evidence of usability. Our key finding is that the XR position feedback game was the most well-received component of the platform, with consistently high SUS usability scores and positive feedback from users. This task, dubbed “Hoop Hustle” in the prototype, required participants to perform wrist movements to control a VR interface with accompanying visual feedback. The strong performance of this task suggests that combining visual feedback in an intuitive pointing game can be highly engaging and easy to use for people with functional weakness. Participants likely benefited from the clear, immediate cause-and-effect in this game, which may have contributed to a sense of accomplishment and control.

In contrast, the VR relaxation module yielded a polarized reaction: some individuals felt deeply relaxed and enjoyed the experience (reflected in very high SUS scores), while others struggled with aspects of the VR environment (leading to poor usability ratings). These divergent outcomes highlight that a one-size-fits-all relaxation experience may not suit everyone; factors such as susceptibility to motion sickness, comfort with wearing a VR headset, and personal preference for meditation-style activities can greatly influence one’s experience. The XR force feedback task showed intermediate and more variable usability. Most participants handled the force-feedback task moderately well (SUS~80‐95 for 4 participants), indicating that they understood the task and could perform it, but one participant (P3) had an extremely negative experience (SUS 27.5). P3’s case is particularly informative: this participant has functional dystonia (a subtype of FND causing involuntary muscle contractions), which likely made it difficult to perform the steady force output required by the task. This resulted in frustration or fatigue, as reflected in both the low usability rating and the participant’s comments describing the force task as “hard to manage” and “tiring.” This finding underscores that individual clinical differences (such as the type of motor symptoms) can dramatically affect the usability of specific training tasks. Notably, the same participant (P3) rated the XR position task very highly (92.5), much higher than they rated the other 2 tasks. We interpret this to mean that while the force feedback task was not well-tolerated by P3, the position feedback game was accessible and enjoyable even for someone with dystonia. It is possible that the position task’s design—emphasizing range of motion and coordination rather than sustained force—was better aligned with this participant’s abilities. This suggests a need for personalized task selection or customization: users might benefit from having multiple training task options and skipping or modifying those that aggravate their symptoms.

Across all tasks, the qualitative feedback provided further insight into these quantitative results. For instance, participants who gave lower SUS scores often cited specific issues that explained their discomfort. Those who rated the VR relaxation poorly mentioned problems like visual graininess and a sense of disorientation when the virtual scene “breaks” (one user described a loss of immersion at a certain transition, eg, reaching a virtual staircase where the illusion was not convincing). On the other hand, participants who enjoyed the relaxation task commented on feeling calm and appreciating the break from active gameplay, which may reflect personal differences in how individuals prefer to engage (active interaction vs passive relaxation). Similarly, mixed feedback on the force task corresponded with whether users felt the haptic feedback was appropriate; some found it novel and motivating, while others found it confusing or difficult to calibrate their strength. We can summarize the qualitative feedback themes as follows.

#### Hardware Comfort and Ergonomics

Multiple participants commented on the VR headset’s weight and fit. One noted that the “headset is heavy” and that the straps were “a bit fiddly” to adjust properly. Another participant suggested the need for a more personalized or lightweight headset, saying they “would prefer [their] own personal headset” if using the system regularly. These comments indicate that physical comfort is a crucial factor, as discomfort could limit how long users with FND (who may have neck or upper body weakness) can wear the device. Ensuring a better fit and lighter hardware in future versions was a unanimous priority among participants.

#### Immersiveness and Visual or Auditory Feedback

Participants generally appreciated the concept of the immersive training tasks, but they pointed out specific issues that broke their sense of immersion. For instance, one participant observed that in the VR relaxation, “the picture quality is bland” (low resolution), which detracted from the experience. Visual artifacts or graphics glitches were noticed by another, who commented that such issues “break immersion.” On the auditory side, a few participants felt the guided meditation voice-over in the VR relaxation was “too artificial” and constant, making it “distracting” rather than soothing. One user recommended incorporating periods of silence or softer, nonverbal audio, noting that “Constant speech is too much–needs time to breathe.” In the XR game, participants enjoyed the sound effects, but one suggested adding more varied sound cues for feedback (eg, different sounds when a hoop is scored versus missed). Enhancing the realism and quality of sensory feedback (both visual and auditory) would likely improve user engagement.

#### Task Difficulty and Personalization

There was a strong consensus on the importance of adjusting the tasks to individual capabilities. In the hoop hustle game, participants had different skill levels; 1 patient with a more severe weakness struggled initially, so the facilitator enlarged the hoop and reduced the required movement range. This kind of on-the-fly personalization was appreciated. Participants explicitly mentioned features they would like to see: “adjustable height [of hoops]” and “hoop size” options, as well as the ability to slow down or speed up the game pace. In the force feedback task, the participant with dystonia noted that the resistance made the task quite challenging for them, but felt it might be helpful if it could be tuned to their strength level. Across the feedback, “personalization” emerged as a key theme–one size does not fit all in this diverse group. Future versions of the platform should include user-specific calibration, difficulty settings, and possibly adaptive algorithms that modify task parameters in real-time based on performance.

#### Perceived Benefits and Engagement

Despite the critiques, most participants expressed enthusiasm for the platform’s concept. Several referred to the approach as a “brilliant idea” and were eager to see it refined. They reported finding the interactive game enjoyable–one health care professional noted that the competitive element of trying to get the ball through the hoop “made it fun, so you forget you’re exercising.” Participants also believed the platform could increase patient motivation to perform rehabilitation exercises, as it “doesn’t feel like therapy” in the traditional sense. The relaxation task was seen as potentially useful for calming down patients before or after physical exercises, although it clearly needs improvement to be effective for everyone.

#### Practical Considerations (Accessibility)

Echoing the Delphi survey results, workshop participants raised practical questions. They debated whether the system would be used in clinics or at home. For home use, participants stressed the need for proper guidance and support: “If this was sent to patients, there would need to be a help guide or 24/7 tech support,” one participant said, concerned about less tech-savvy users. The idea of a shared device versus personal ownership was discussed; some felt a single headset could be used by multiple patients in a clinic if properly sanitized, while others thought long-term users would benefit from having their own device configured to their needs. Concerns about cost were mentioned again; one participant estimated “it’s [£]1000… (US $1330) I could not afford [this]” and hoped it would be provided through the NHS or insurance. These discussions highlight that for the platform to be implementable, issues of cost, training, and technical support must be addressed alongside its technical development.

These thematic insights demonstrate the value of a mixed methods approach: the quantitative data identified where usability was strong or weak, and the qualitative data helped explain why those outcomes occurred. Crucially, the workshop confirmed that co-design is not only feasible but beneficial in developing neurotechnology for FND. Participants’ real-time feedback led us to identify specific improvements (eg, modifying the VR content and adding adjustable settings in the game) that we might not have fully appreciated without their involvement. The inclusion of both patients and clinicians ensured that the usability assessment considered practical use in a clinical context.

### Comparison with Previous Work

Our findings align with existing literature emphasizing user-centered design for health technologies. Previous studies have noted that even small samples (5‐10 users) can uncover the majority of usability issues in a system [29]. In our case, 6 users were sufficient to highlight distinct strengths and weaknesses of the platform. The variability in VR relaxation feedback is reminiscent of observations in broader VR applications: while VR can provide immersive therapeutic experiences, factors like motion sickness and comfort remain challenges to address. The need for personalization in rehabilitation technology is well-documented; for instance, usability studies of other rehab games have found that adaptive difficulty can significantly improve user engagement and outcomes. Our results specifically extend this understanding to FND, suggesting that personalization may not only improve engagement but might be necessary to accommodate neurological symptoms like dystonia or fatigue. From a neurological perspective, the concept of using haptic feedback and VR to retrain the perception-action cycle in FND draws on theories of sensory attenuation and agency in functional movement disorders. By providing congruent visual and haptic inputs corresponding to the user’s intended movements, the platform aims to reinforce the association between effort and sensory feedback, potentially strengthening the efference copy mechanism that is hypothesized to be underactive in patients with FND [14,30,31]. While our study did not directly measure clinical outcomes or neurophysiological changes, the positive usability of the position and force feedback tasks is a critical first step toward implementing such therapeutic concepts in practice. A recent review by [12] also emphasized VR’s promise for addressing mechanisms of agency and attention in FND; our practical findings complement this by showing that patients are willing to engage with VR or haptic systems, provided they are comfortable and accessible.

### Limitations

This study has several limitations. First, the sample size was small (5‐6 participants for usability testing), and all participants were from a single clinical center and also coauthors, which could introduce some bias or limit critical feedback. The findings should be interpreted as preliminary and exploratory; a larger, independent sample will be needed to validate and generalize the usability results. Second, participants’ familiarity with XR technology varied, and those with previous VR or gaming experience might have found the system easier to use, potentially influencing their SUS scores. We did not formally quantify each participant’s XR technology background, which is a confounding factor that future studies should measure. Third, we focused on 3 specific XR tasks (VR relaxation, XR position feedback, and XR force feedback). Other functionalities (eg, bilateral training or cognitive tasks in XR) were not included and could present additional usability challenges or benefits not captured here. Fourth, the reliance on subjective SUS scores introduces potential bias, as individual expectations or novelty effects can influence ratings. We mitigated this by collecting detailed qualitative feedback, but objective performance metrics were not analyzed in this pilot. Fifth, as an initial co-design and usability study, we did not assess clinical efficacy, for instance, whether using the platform yields improvements in motor function or FND symptoms. Such outcomes will need evaluation in subsequent trials. Finally, our personalization of the tasks was done manually by the facilitators rather than through built-in adaptive algorithms. This limits the consistency of the user experience; an automated personalization mechanism would be ideal to ensure each user gets an optimally challenging experience. Despite these limitations, the study provides valuable insights into the user experience of an XR neurotechnology platform tailored for FND. To our knowledge, this is one of the first studies to report detailed usability data for an XR haptics platform in FND rehabilitation. The co-design approach proved effective in identifying user priorities and potential pitfalls early in the development process.

### Future Directions

**Figure 3.**
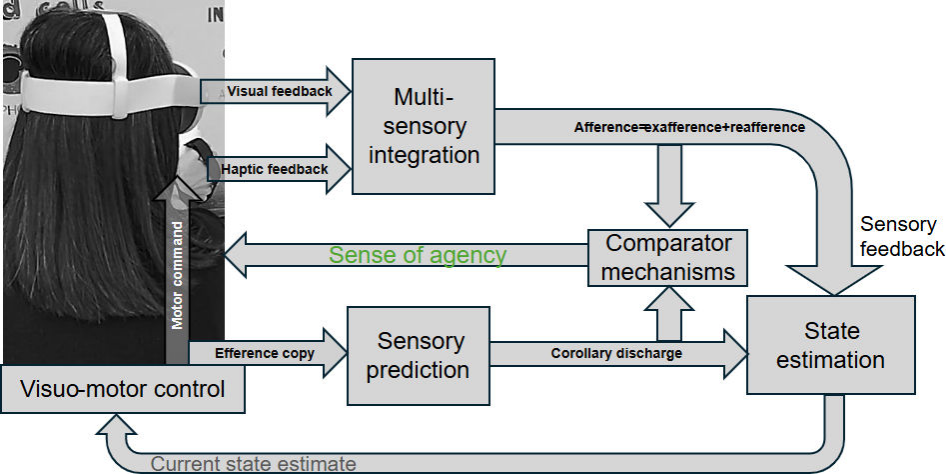
Based on the comparator model, when a motor command is issued, an accompanying efference copy is generated, which allows the brain to predict the expected sensory outcome of the action. This predicted outcome is then compared to the actual sensory feedback upon action completion. A strong the feeling of agency is experienced if there is a close match between predicted (corollary discharge) and actual sensory information (afference) from the environment. This comparator model can also explain feeling of agency in virtual extended reality (XR) environments where a virtual representation mimics the user’s physical movement, providing exafference that, when combined with reafference, provides users the sense of agency (feeling of agency).

The next steps following this study will address the identified issues and test the platform on a broader scale at home [32]. FMD involves involuntary-feeling but voluntary-appearing movements, linked to a disrupted sense of agency due to impaired sensory attenuation, that is, the brain’s predictive suppression of self-generated sensory feedback [33]. This impairment, involving brain regions like the primary motor cortex, cerebellum, and right temporo-parietal junction, leads to difficulties in distinguishing self-initiated actions from external stimuli [34]. Conversely, sensory amplification, mediated by the posterior parietal cortex, heightens sensory perception through attention. XR presents therapeutic potential by balancing sensory attenuation and amplification [6]. VR allows controlled manipulation of predictive coding, helping recalibrate agency and sensory processing in FMD. The comparator model (refer to Figure 3) suggests that agency arises when predicted sensory outcomes align with actual feedback, which can be reinforced through haptic feedback in XR. Here, linking active inference in motor control lies in its ability to explain and address motor dysfunctions [35]. By recognizing that the brain updates perceptions and modifies actions to minimize prediction errors, this framework offers insights into abnormal motor control, where disrupted sensory prediction leads to impaired agency and movement errors. In rehabilitation, this perspective supports the development of XR biofeedback interventions, where haptic and visual feedback can help recalibrate faulty sensorimotor predictions. By reinforcing accurate sensory-motor associations, these technologies may restore agency and improve motor function, offering a novel, personalized approach to therapy. Indeed, XR technologies have been shown to enhance sensorimotor processing, but usability for patients with FMD must be assessed. Early user involvement, particularly in conditions like functional dystonia, is critical to refining XR rehabilitation design. Our study engaged stakeholders from academia, industry, and health care (NHS, England) to classify technological needs into incremental or revolutionary advancements. Notably, no commercial or research-based XR biofeedback systems currently exist specifically for FMD rehabilitation.

In response to user feedback, we are working with the developers to improve the VR relaxation module (enhancing graphics, refining the audio guidance, and possibly adding options for different scenes or background music) [32]. We are also implementing in-software settings that allow end-users or therapists to easily adjust game difficulty, visual or auditory feedback levels, and force feedback intensity. In addition, we plan to incorporate a brief calibration or tutorial at the start of a session, where the system can gauge a user’s comfortable range of motion and strength, and automatically set initial task parameters accordingly. These changes aim to embed personalization directly into the platform. A follow-up study is being designed to involve a larger cohort of patients with FND in a multisession at-home trial with the refined platform [32]. That study will evaluate not just usability, but also short-term effects on motor function and symptoms, using clinical scales and objective performance metrics within the game. We will also examine learning effects–whether repeated use leads to improved user proficiency or changes in feedback preferences–to understand how usability evolves over time. An important future direction is to explore remote or home usability of this platform [32]. Given the interest in home-based rehabilitation (and lessons learned during the COVID-19 pandemic), we aim to test whether patients can effectively use the XR system at home with minimal supervision. This will involve developing comprehensive user guides, integrating remote monitoring capabilities (so therapists can track usage and progress), and ensuring robust technical support is available. Addressing these factors will be essential for translating this coproduced XR platform into a scalable, real-world therapeutic option for individuals with FND.

### Conclusions

Through a collaborative coproduction approach, we developed and pilot-tested a novel XR (VR+ haptic) biofeedback training platform for patients with functional upper limb weakness due to FND. Our usability findings are encouraging: an interactive XR position feedback game was rated highly usable by all participants, and a VR relaxation experience received very positive feedback from some users. At the same time, the variability in responses, particularly the challenges faced by one participant during the force feedback task, highlights the necessity of a flexible, user-tailored design in such neurotechnologies. One-size-fits-all solutions are unlikely to succeed in the FND population given the diversity of symptoms and user preferences. By systematically incorporating user feedback, we identified concrete areas for improvement (such as hardware comfort and software adaptability) that will guide the next iteration of the platform. This study demonstrates that patients with FND and clinicians are not only capable of providing meaningful input into technology design but are eager to do so when the goal is to enhance therapy. With further refinement and larger-scale testing, the XR platform has the potential to become a valuable tool in FND rehabilitation, offering engaging, at-home training that reinforces patients’ agency and motor function in a way that is enjoyable and customized to their needs.

## Supplementary material

10.2196/68580 Multimedia Appendix 1 NRC Rehabilitation Technologies Conference 2024 poster.

10.2196/68580 Multimedia Appendix 2 NRC Rehabilitation Technologies Conference 2024 slides.

10.2196/68580 Multimedia Appendix 3 Human Robotix’s HRX-1 system.

10.2196/68580 Multimedia Appendix 4 Nudge Reality’s XR games.

10.2196/68580 Multimedia Appendix 5 XR System Usability testing script.

10.2196/68580 Multimedia Appendix 6 XR System Usability testing results – Force Feedback.

10.2196/68580 Multimedia Appendix 7 XR System Usability testing results – PositionFeedback.

10.2196/68580 Multimedia Appendix 8 XR System Usability testing results – Relaxation.

## Abbreviations

FMD: functional motor disorder
FND: functional neurological disorder
GDPR: General Data Protection Regulation
HIPAA: Health Insurance Portability and Accountability Act
HRX-1: human robotix system
MR: mixed reality
NHS: National Health Service
NRC: National Rehabilitation Centre
PPIE: Patient and Public Involvement and Engagement
SUS: system usability scale
VR: virtual reality
XR: extended reality
