# Supplementary material for: Extended Reality Biofeedback for Functional Upper Limb Weakness: Mixed Methods Usability Evaluation
Source: JMIR XR Spat Comput. 2025 Jun 3;2:e68580. doi: 10.2196/68580 (PMC12671321; doi:10.2196/68580)
Supplement: Multimedia Appendix 1 [file xr-v2-e68580-s001.pdf]

VR Haptics Biofeedback Training for Functional Limb Weakness: Insights from the First Round of a Delphi Survey

Abhijit Das (Royal Preston Hospital), Matthew Newsham (patient advocate), Katerina Hatjipanagioti (patient advocate), Alastair Buchanan (Nudge Reality), Ildar Farkhatdinov (King's College London), Anirban Dutta (University of Birmingham)

Presenting author: Matthew Newsham

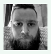

Correspondence: a.dutta.1@bham.ac.uk

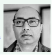

UNIVERSITY OF BIRMINGHAM

Highlights

- VR haptics biofeedback training shows significant potential for limb rehabilitation in FND patients.
- Immersive and customizable VR experiences, combined with haptic feedback, offer a novel approach to improving motor control.
- Challenges include the high cost of equipment, accessibility concerns, and usability issues like VR motion sickness.
- Next steps involve refining the technology based on feedback and making it more accessible to a broader population of FND patients.

Methods

- An online Delphi survey, designed to gather feedback from Expert by Experience (EbyE) – individuals with lived experience – was conducted.
- Initial round involved 20 participants, all diagnosed with FND. The survey's participants were predominantly female, with a peak age range of 35 to 44 years.
- Participants were asked about their familiarity with VR technology and haptic feedback, as well as their perceptions of its applicability in biofeedback training for functional limb weakness.
- The Delphi method aimed to gather collective to help refine VR Haptics Biofeedback technology for better usability.

Background

FND is a condition characterized by involuntary neurological symptoms, including seizures, sensory disturbances, and limb weakness. These symptoms pose significant rehabilitation challenges.

Traditional physiotherapy approaches often fall short [1], necessitating innovative solutions to improve patient outcomes.

Virtual Reality (VR) technology, combined with haptic feedback (the use of tactile sensations to replicate touch) in a mixed reality approach, offers a new frontier for rehabilitation.

By providing real-time, immersive biofeedback, VR with haptics biofeedback training has the potential to enhance motor control and limb rehabilitation in FND patients in home settings.

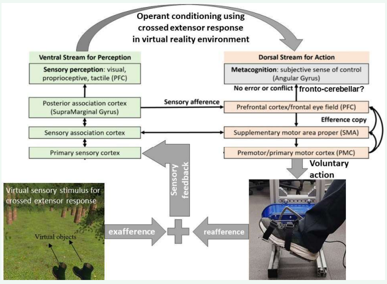

Participant Demographics

Participants included individuals clinically diagnosed with FND. Functional neurologic disorder (FND) involves genuine, involuntary neurological symptoms and signs, such as seizures, weakness, and sensory disturbances, which have distinctive clinical features. We present the results from our first round of the Delphi survey, aimed at translation of the Virtual Reality (VR) with haptics technology for biofeedback training in FND. The survey gathered online feedback from twenty (N=20) individuals with lived experience of FND considered experts for technology translation.

The online survey data collected, including age and gender, showed a peak in the 35-44 years age bracket and a predominance of females

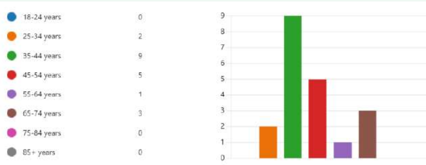

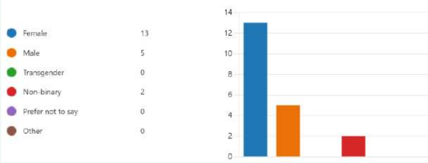

Experience of VR Haptics Technology

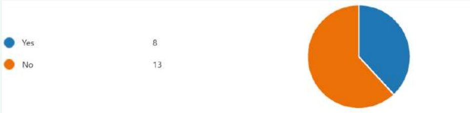

Perception of VR Haptics Technology

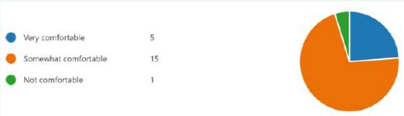

A significant portion of participants were aware of VR technology, though the depth of knowledge varied. Experience with haptic feedback technology was less common, as estimated from the comments. Key features highlighted in the comments included customizable exercises, real-time feedback, and immersive environments. Participants generally perceived VR technology to be comfortable, though one expressed reservation about the mask/goggles form. There was less experience with haptic feedback technology estimated from the comments.

ID	Name	Responses
1	anonymous	used both recreationally and in a vestibular therapy setting
2	anonymous	Flight simulation, gaming.
3	anonymous	It was a simple underwater VR where I could move around an look
4	anonymous	A friends meta quest gaming.
5	anonymous	A brief go at a driving vr game
6	anonymous	A fun immersive experience. However, playing for too long makes me feel motion sick.
7	anonymous	We own a VR head set at at home used for entertainment
8	anonymous	My son has a virtual reality oculus device. So have used it a couple times. Also the science museum that we go to often have some virtuality areas.

ID	Name	Responses
1	anonymous	I imagine it would be a mask/goggles of some sort so might feel claustrophobic. It make make me feel nauseous as I can't watch some video games due to the movement.

Cybersickness, the "elephant in the room": Cybersickness is a form of motion sickness that arises from immersive eXtended Reality (XR) environments, such as virtual and augmented reality (VR/AR), with symptoms ranging from mild discomfort to nausea and, in rare cases, an emetic response. It affects 20%-95% of users, with common symptoms including headaches, eyestrain, and disorientation. VR typically leads to disorientation and nausea, while AR causes oculomotor issues like eyestrain. VR's symptoms are more noticeable, causing users to limit exposure, whereas AR's subtler effects may lead to prolonged use, raising safety concerns. Research is needed to better understand how XR environments contribute to cybersickness and how to mitigate its effects..

Awareness of VR Haptics Technology for Biofeedback

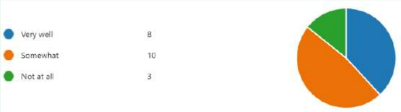

Relevance and Impact of VR Haptics Technology

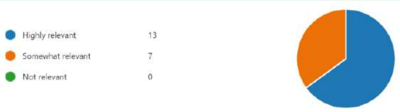

Rehabilitation Priority

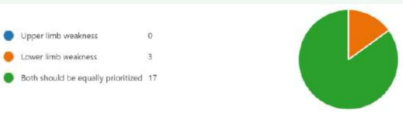

Potential Benefits

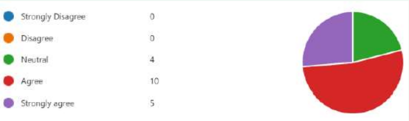

Positive Perception of VR Haptics: Participants overwhelmingly agreed on the relevance of VR haptics for functional limb rehabilitation, particularly in improving motor control for both upper and lower limbs. The customizable nature of VR environments, combined with haptic feedback, was recognized as a potential game-changer for rehabilitation exercises. Participants appreciated the immersive experience and real-time biofeedback, which can improve engagement and recovery outcomes.

Barriers and Challenges

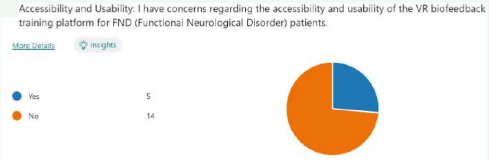

ID	Name	Responses
1	anonymous	How would you get the equipment? How do you know if you're using it correctly? What if you encounter difficulties using it?
2	anonymous	Not everyone's abilities with vr come naturally some people cannot use it at all. So if this rehabilitation technique works how will those people access this? Also would this be private and have a large cost? Or will this be available with the NHS? As I know personally I could not afford the system itself nor the programe
3	anonymous	VR is very hard to understand for many people,
4	anonymous	This might be just me but the motion sickness is always worse for me, whilst gaming, when my FND symptoms are more intense than normal
5	anonymous	Equipment and WiFi might be limited for some people. Neurologists don't always offer their patients treatment that is outside the norms.

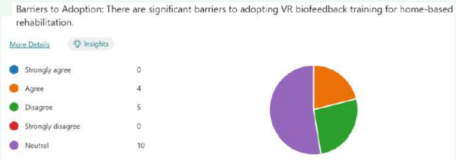

ID	Name	Responses
1	anonymous	Lack of support in general/No access to such things.
2	anonymous	Equipment and WiFi might be limited for some people. Insurance may not cover it.
3	anonymous	There could be a safety issue if falling, Overstimulation so could cause seizures

Barriers to Adoption: Despite the positive outlook, several challenges were identified that could limit the widespread adoption of VR haptics training.

- Cost: The high price of VR and haptic equipment was seen as a significant barrier, especially for patients reliant on public healthcare services or without the means to afford such technology.
- Accessibility: Participants expressed concerns about whether the technology would be made widely available through public health systems like the NHS or if it would remain a costly private treatment.
- Usability: Many participants voiced discomfort with VR systems, with some experiencing motion sickness, particularly when their FND symptoms were more intense. Additionally, concerns were raised about the learning curve for patients unfamiliar with VR technology, and whether sufficient technical support would be available to assist them.
- Overstimulation: A considerable portion of participants reported sensory overload while using VR, which could hinder its adoption, especially for those with heightened sensitivity due to FND.

Conclusion

Participants Promise of VR with Haptics in FND Rehabilitation: Overall, participants viewed VR haptics as a promising tool for enhancing rehabilitation in individuals with FND.

However, significant barriers need to be addressed before the technology can be implemented widely. The cost of the equipment, accessibility, and concerns about usability must be overcome for VR haptics to be a practical solution for rehabilitation.

Although our survey's small sample size is a limitation, a theoretical framework can still guide the refining of design requirements [2].

Next Steps: Future rounds of the Delphi survey will focus on refining design requirements for the VR haptics system based on feedback from same participants.

Key areas for improvement include reducing the motion sickness associated with VR usage with Augmented Reality [3], cost-effectiveness analysis of the equipment, and ensuring that the technology is accessible to patients with varying levels of technical proficiency. Further research will also investigate how the system can be tailored to meet the needs of patients with more severe FND symptoms.

References

[1]. Specialist physiotherapy for functional motor disorder in England and Scotland (Physio4FMD): a pragmatic, multicentre, phase 3 randomised controlled trial. Nielsen, Glenn, Beaves, Emily et al. The Lancet Neurology, Volume 23, Issue 7, 675 – 686.

[2]. Virtual reality in functional neurological disorder: a theoretical framework and research agenda for use in the real world. Brouwer D, Morrin H, et al. BMJ Neurol Open. 2024 Jul 5;6(2):e000622.

[3]. Virtual, augmented, mixed, and extended reality interventions in healthcare: a systematic review of health economic evaluations and cost-effectiveness. Gómez Bergin, A.D., Craven, M.P. BMC Digit Health 1, 53 (2023).
