# Supplementary material for: Extended Reality Biofeedback for Functional Upper Limb Weakness: Mixed Methods Usability Evaluation
Source: JMIR XR Spat Comput. 2025 Jun 3;2:e68580. doi: 10.2196/68580 (PMC12671321; doi:10.2196/68580)
Supplement: Multimedia Appendix 2 [file xr-v2-e68580-s002.pdf]

NRC Rehabilitation Technologies Conference

Co-production of a platform technology for VR biofeedback training under operant conditioning for functional lower limb weakness

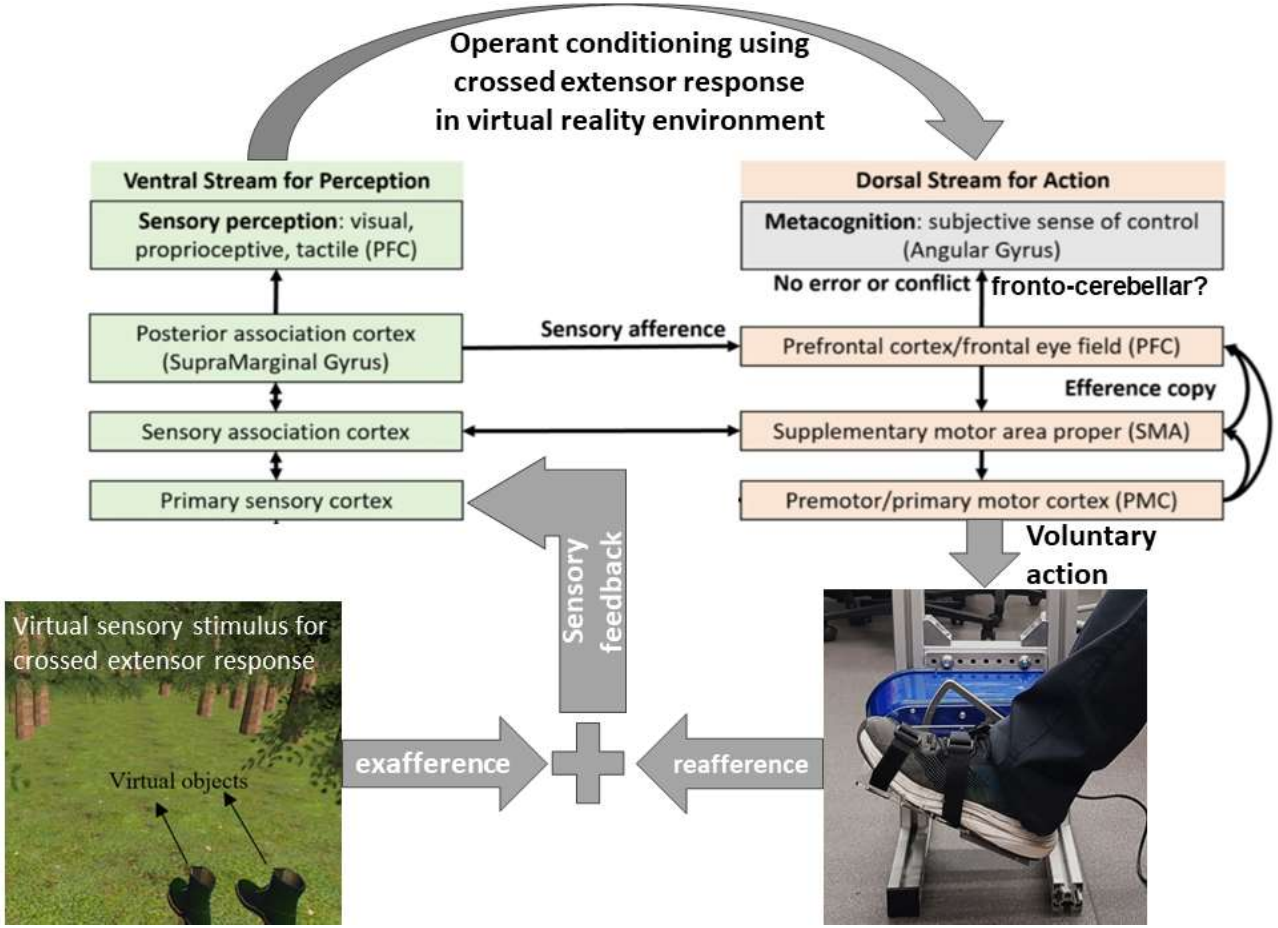

18 September 2024

University of Birmingham

Lead partners

Rehab Technologies Network | Loughborough University | University of Nottingham UK | CHINA | MALAYSIA | NRC | NHS Nottingham University Hospitals NHS Trust

Funded by

UKRI Engineering and Physical Sciences Research Council

Agenda

- Introduction to Project and Pre-Design
- Overview of VR and Haptics System Used for Co-production
- First-Round Delphi Survey Results
- U.S. FDA Guidelines and Cost-effectiveness Analysis
- Next Delphi Round

Rehab Technologies Network
Loughborough University
University of Nottingham UK | CHINA | MALAYSIA
NRC
NHS Nottingham University Hospitals NHS Trust
Funded by UKRI Engineering and Physical Sciences Research Council

INTRODUCTION TO THE BACKGROUND

BIOFEEDBACK TRAINING

Introduction

"Biofeedback is a mind–body technique in which individuals learn how to modify their physiology for the purpose of improving physical, mental, emotional and spiritual health." Frank DL, Khorshid L, Kiffer JF, Moravec CS, McKee MG. Biofeedback in medicine: who, when, why and how? Ment Health Fam Med. 2010 Jun;7(2):85-91.

"During biofeedback training, sensors attached to your body detect changes in your pulse, skin temperature, muscle tone, brain-wave pattern or some other physiological function. These changes trigger a signal a sound, a flashing light, and a change in pattern on a video screen that tells you that the physiological change has occurred." https://www.health.harvard.edu/

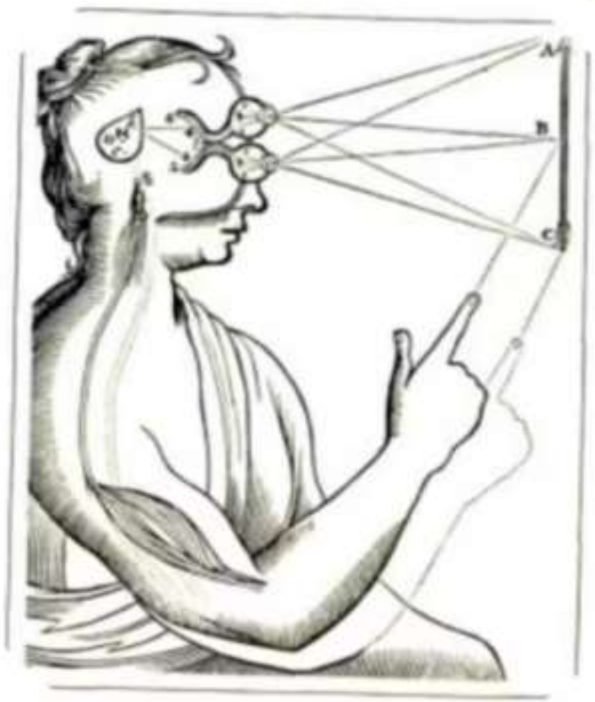

Rehab Technologies Network | Loughborough University | University of Nottingham UK | CHINA | MALAYSIA | NRC | NHS Nottingham University Hospitals NHS Trust | UKRI Engineering and Physical Sciences Research Council

INTRODUCTION TO THE PROJECT

NRC Rehabilitation Technologies Conference

- Sensory afference can arise from external events (**exafference**) and our own actions (**reafference**).
- **Comparator model** (cerebellum) posits that disparity between sensory afference, and the predicted sensory input (efference copy) forms a sensory prediction error.
- **Implicit recalibration** for building subjective sense of control is influenced by sensory prediction errors, which may be modulated with affected limb movement feedback in the VR with haptics in a mixed reality setting for Operant Conditioning.

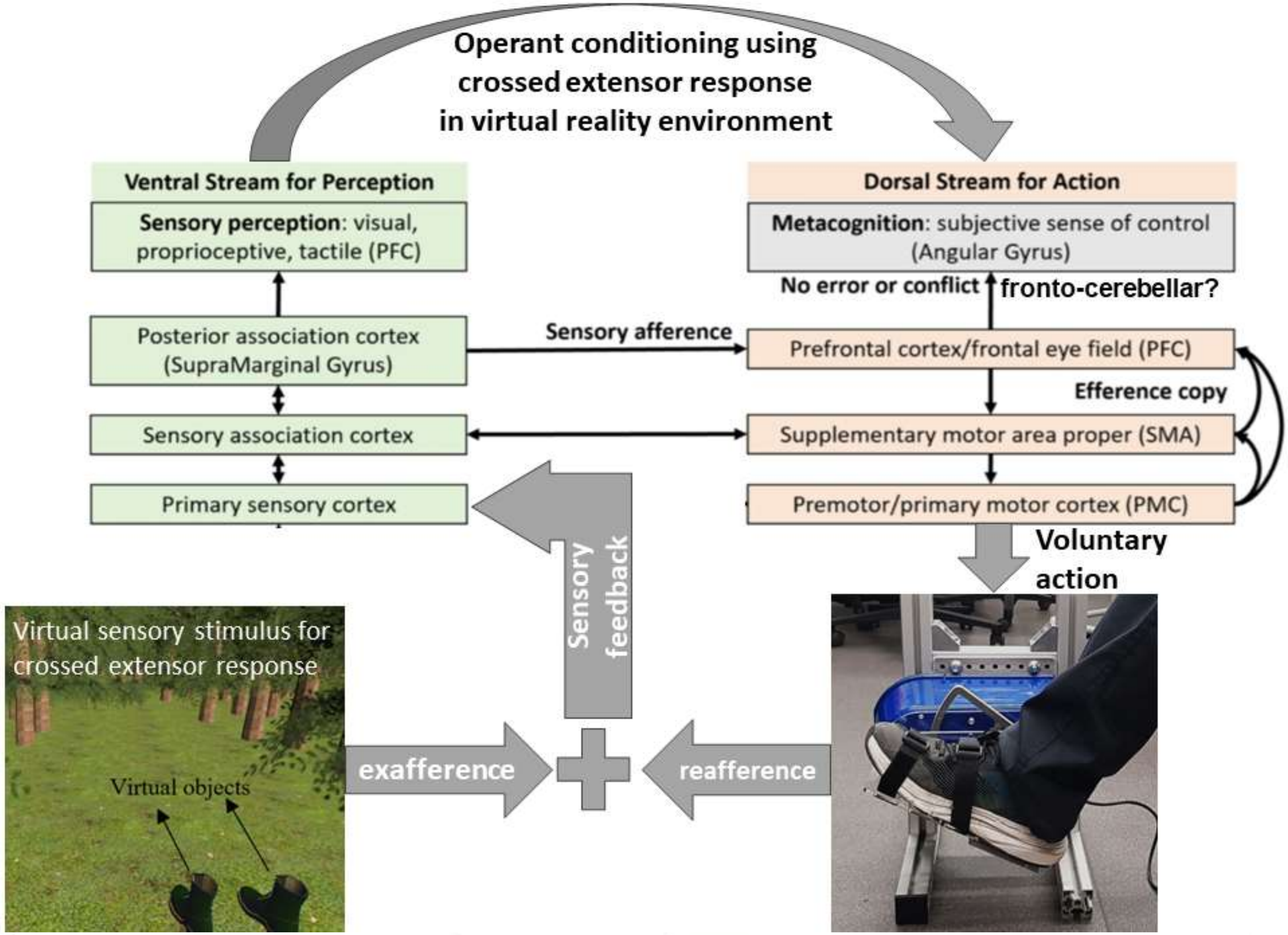

Rehab Technologies Network | Loughborough University | University of Nottingham UK | CHINA | MALAYSIA | NRC | NHS Nottingham University Hospitals NHS Trust | UKRI Engineering and Physical Sciences Research Council

PRE-DESIGN OUTCOME

Co-creation of digital health neuro technologies for management of functional neurological disorders (FND): a workshop

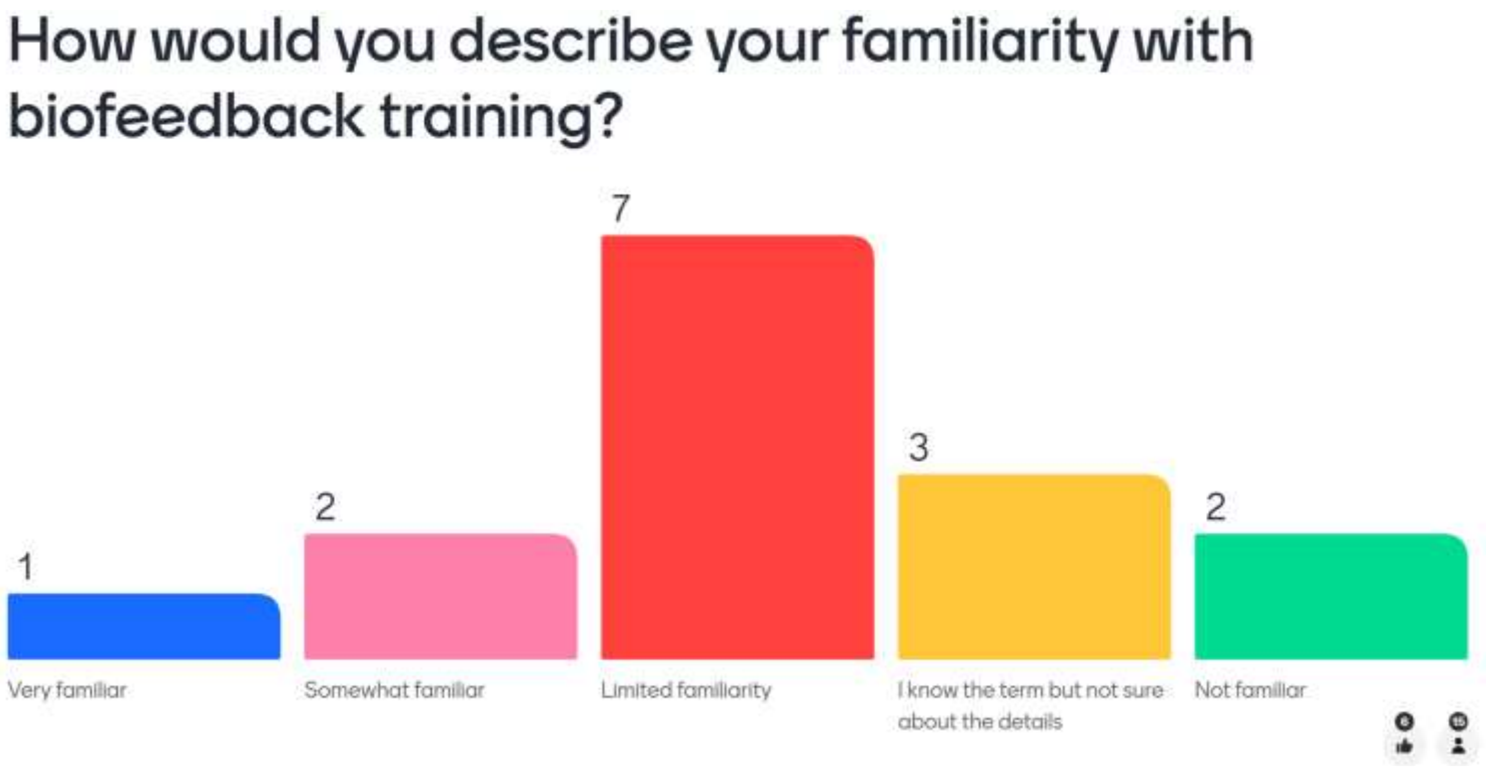

Rehab Technologies Network
Loughborough University
University of Nottingham UK | CHINA | MALAYSIA
NRC
NHS Nottingham University Hospitals NHS Trust
Funded by
UKRI Engineering and Physical Sciences Research Council

PRE-DESIGN OUTCOME

Co-creation of digital health neurotechnologies for management of functional neurological disorders (FND): a workshop

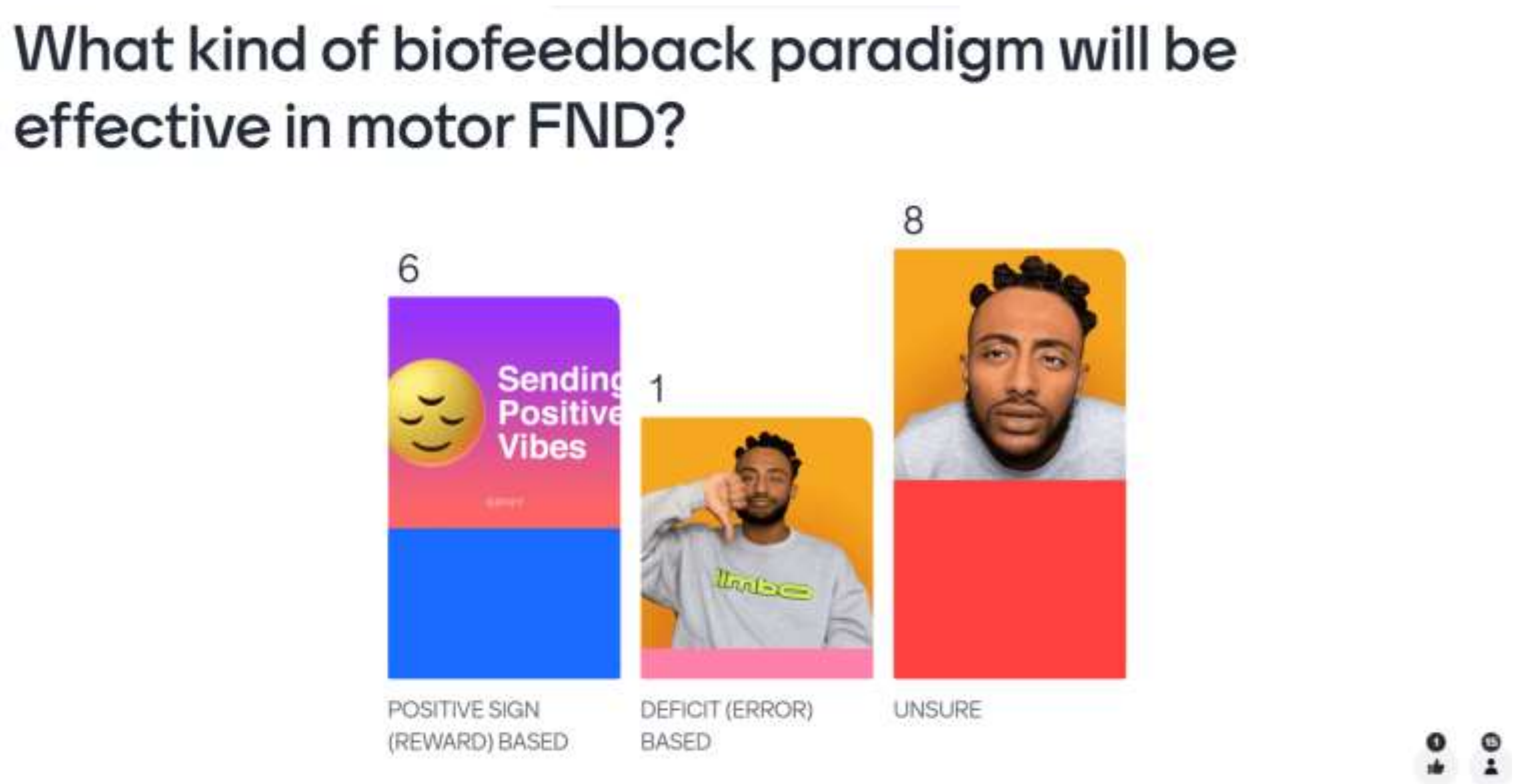

DESIGN GOAL: GENERATIVE PROCESS

Functional weakness home-based rehabilitation

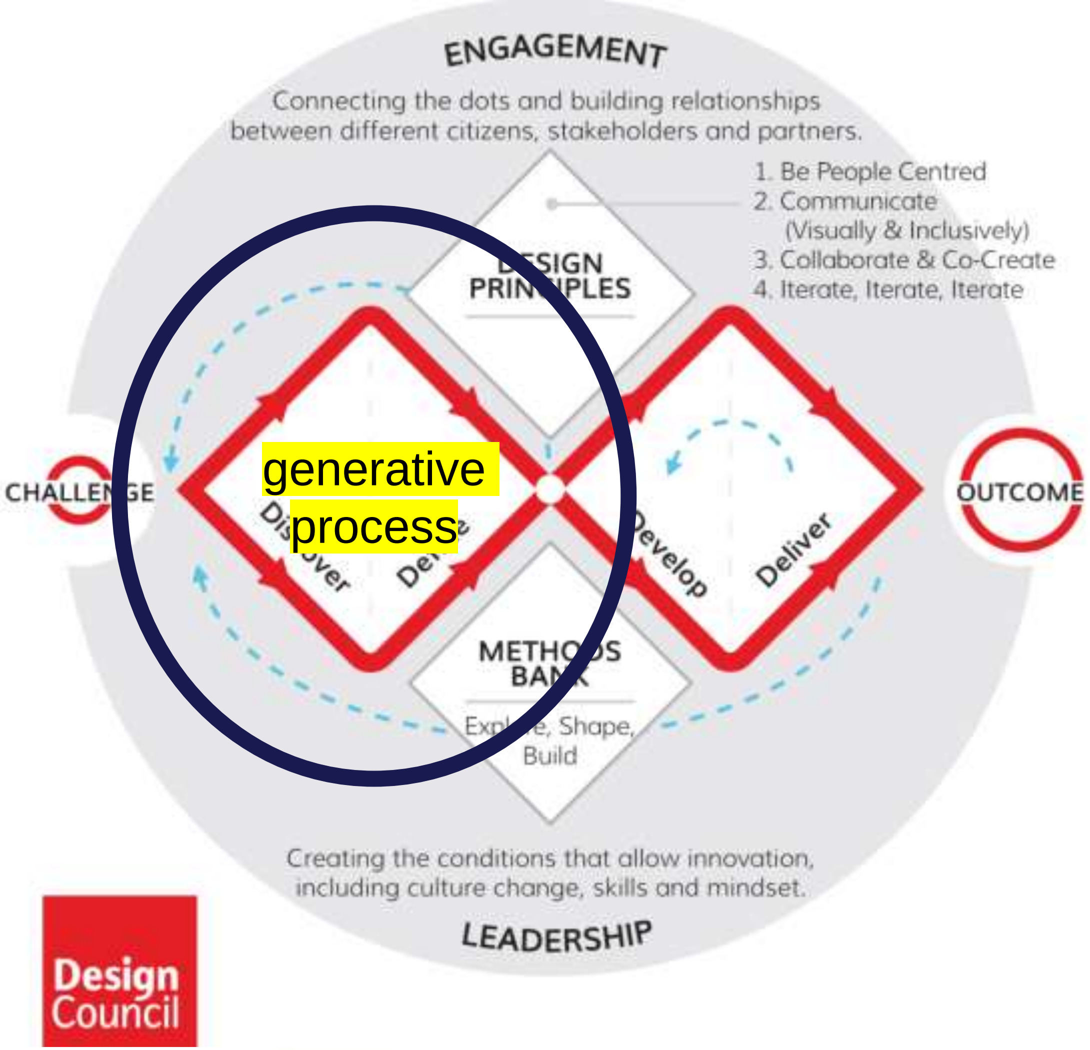

generative process

Design requirements

Funded by

OVERVIEW: INNOVATION vs INVENTION

Generative process

Movement rehabilitation

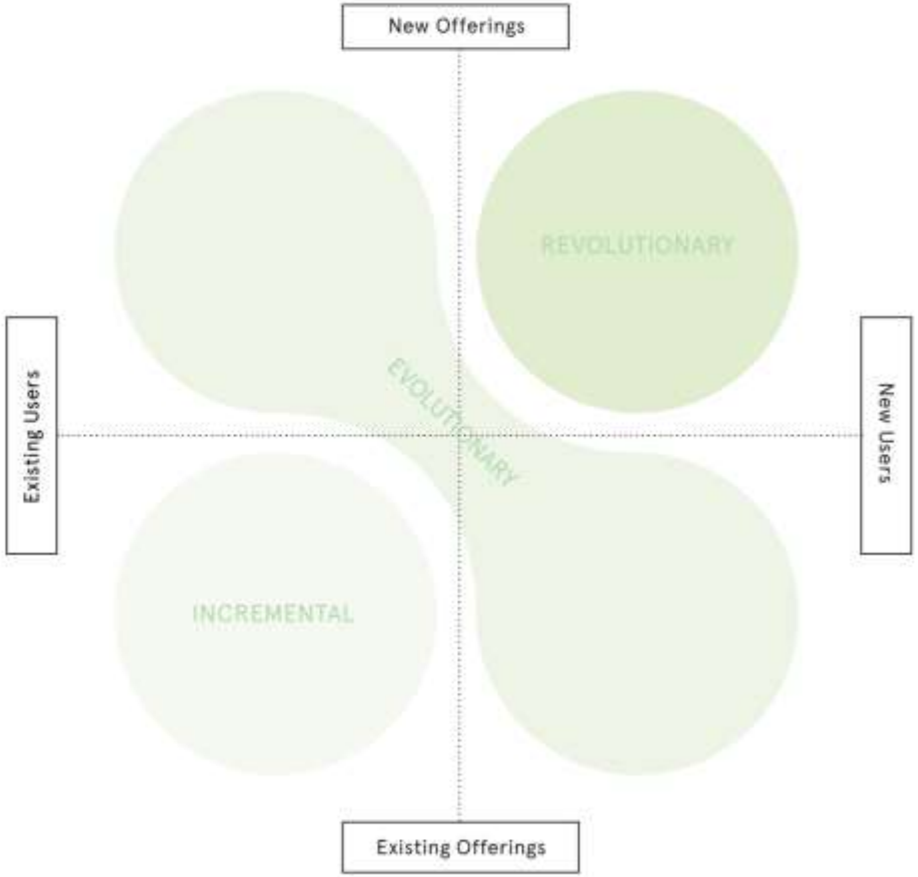

Motor FND rehabilitation

Funded by

EXISTING HRX-1 (HUMAN ROBOTIX)

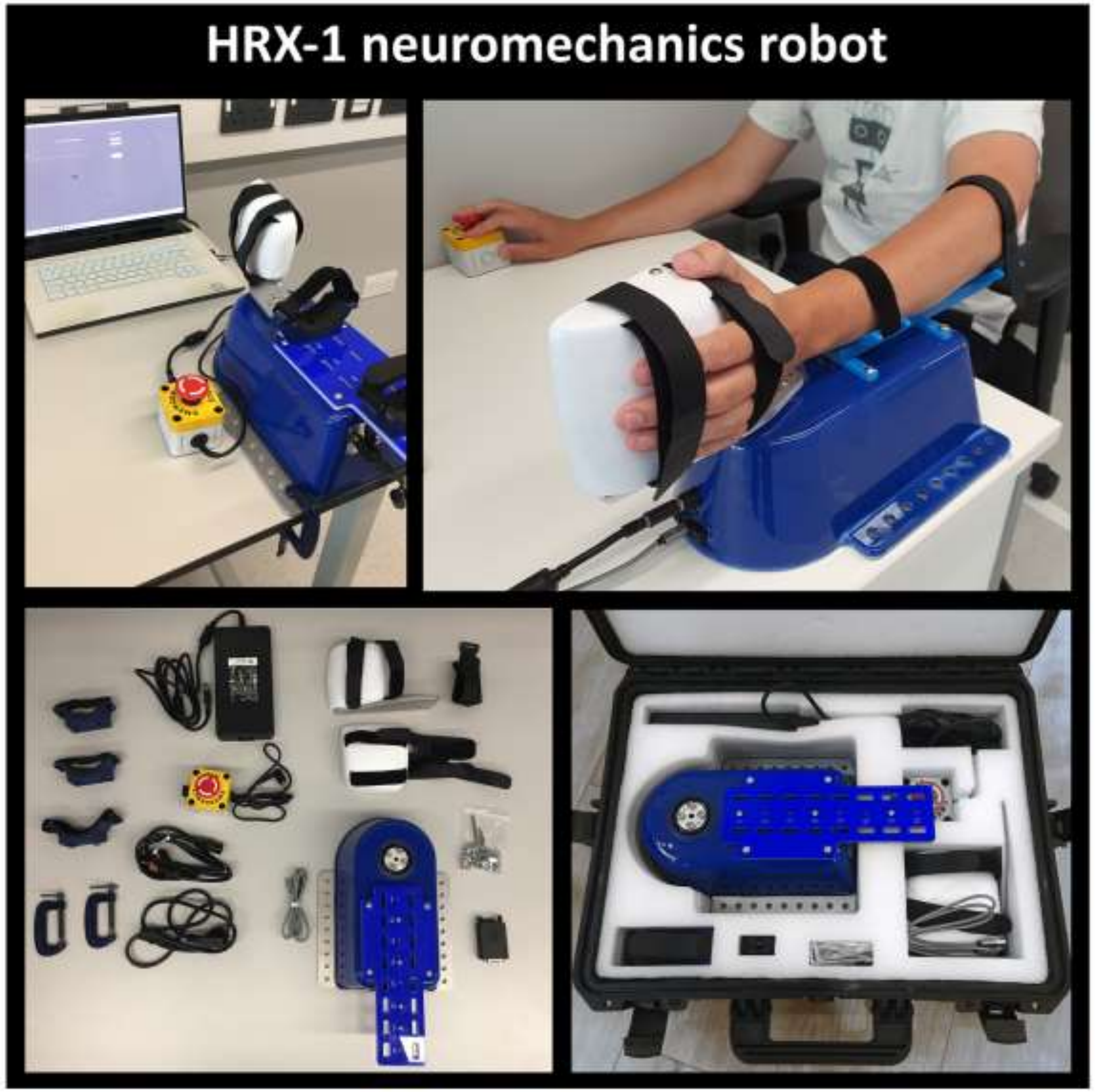

Rehab Technologies Network
Loughborough University
University of Nottingham UK | CHINA | MALAYSIA
NRC
NHS Nottingham University Hospitals NHS Trust
Funded by
UKRI Engineering and Physical Sciences Research Council

EXISTING VR GAMES (NUDGE REALITY)

Game Concepts

1. Hoop Hustle

"Hoop Hustle" is a reaction-based game that tests precision, timing, and skill. Players twist their wrist to control a basketball hoop along an arc beneath ball droppers, catching balls as they fall. The game's difficulty increases with speed and distance, enhancing reflexes and control.

2. Ring Master

In "Ring Master," players use a wrist-twisting motion to control a claw that places coloured rings on target poles. The game combines strategy and quick reactions, with progressive difficulty and special rings adding complexity. The goal is to match three or more rings of the same colour to score points.

3. Twist'n'Ring

"Twist'n'Ring" immerses players in a VR environment where they throw rings at targets using wrist twists. The game challenges players to master precision and technique, with progressive levels introducing dynamic obstacles and environmental factors.

These games not only make rehabilitation engaging but also provide a measurable way to track progress and improve arm movement in FND patients. Each game leverages realistic physics, intuitive controls, and vibrant visuals to create an immersive and therapeutic experience.

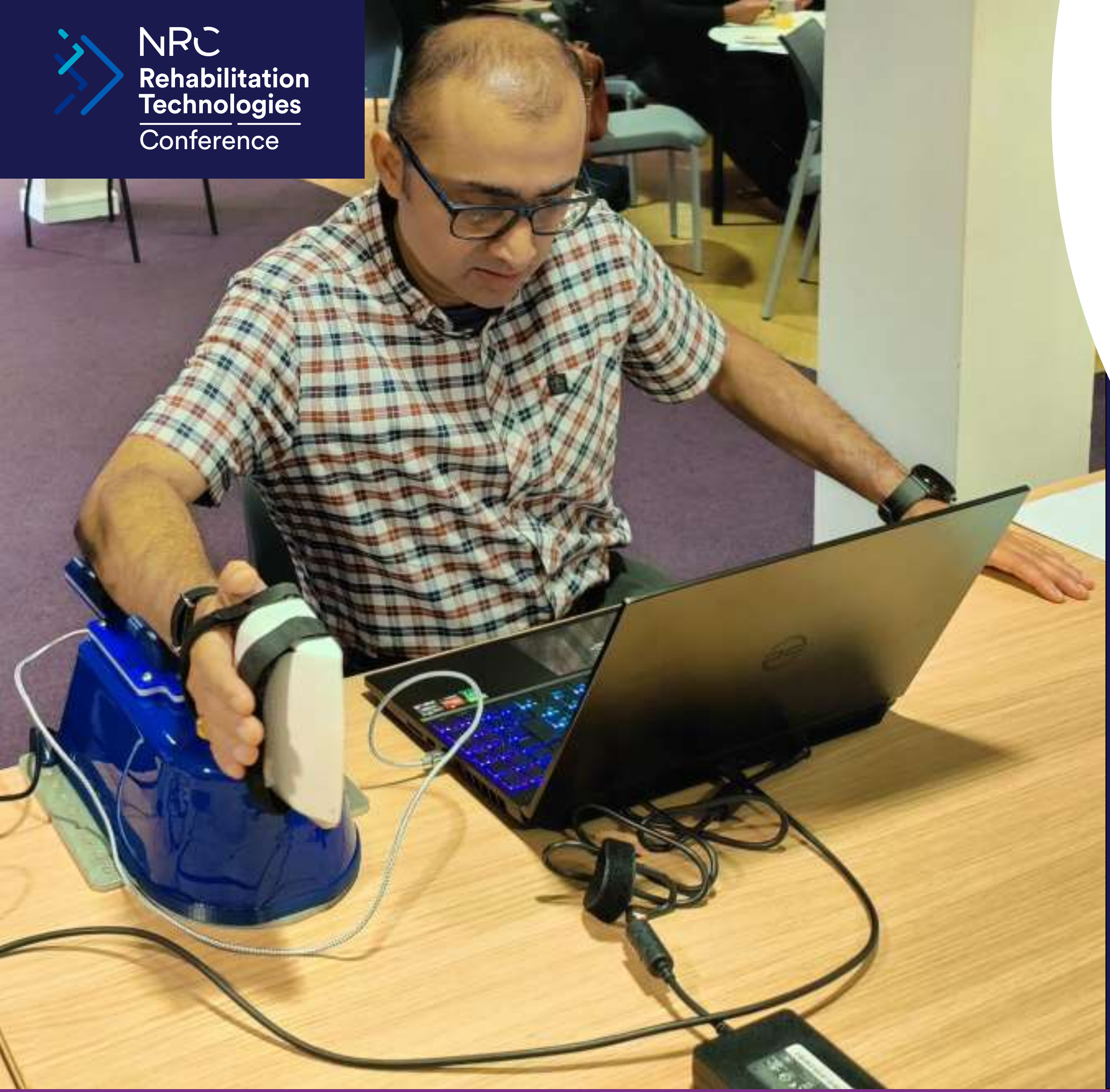

NRC Rehabilitation Technologies Conference

FIRST ROUND DELPHI SURVEY

VR WITH HAPTICS BIOFEEDBACK TRAINING

ONLINE SURVEY PARTICIPANTS (N=20)

NRC Rehabilitation Technologies Conference

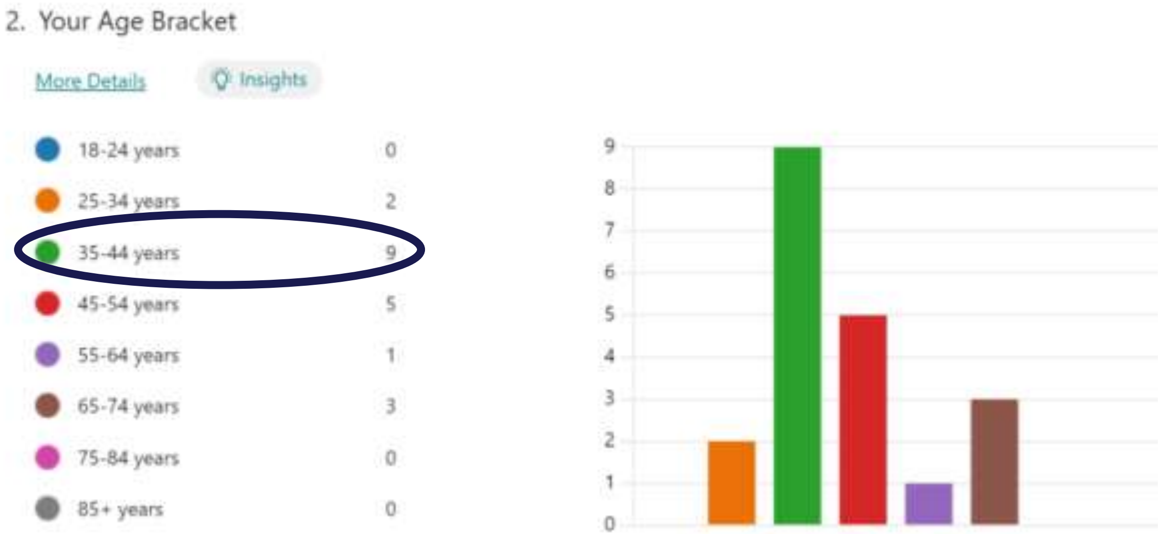

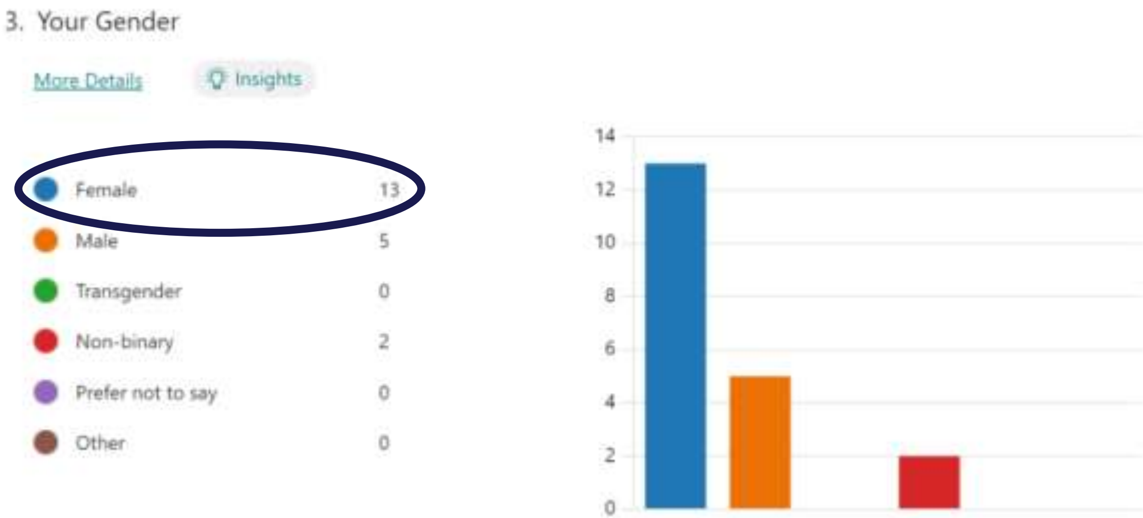

Participants included individuals clinically diagnosed with FND. Functional neurologic disorder (FND) involves genuine, involuntary neurological symptoms and signs, such as seizures, weakness, and sensory disturbances, which have distinctive clinical features.

First round of the Delphi survey collected online feedback from **twenty (N=20) individuals with lived experience of FND** considered experts by experience (EbyE) for the usability of Virtual Reality (VR) with haptics technology for biofeedback training in FND.

The online survey data collected, including age and gender, showed a peak in the 35-44 years age bracket and a predominance of females

Rehab Technologies Network
Loughborough University
University of Nottingham UK | CHINA | MALAYSIA
NRC
NHS Nottingham University Hospitals NHS Trust
Funded by
UKRI Engineering and Physical Sciences Research Council

SURVEY RESULTS FROM DELPHI ROUND 1

NRC Rehabilitation Technologies Conference

- **Positive Perception of VR Haptics**: Participants overwhelmingly agreed on the relevance of VR with haptics for functional limb rehabilitation, particularly in improving motor control for both upper and lower limbs. The customizable nature of VR environments, combined with haptic feedback, was recognized as a potential game-changer for rehabilitation exercises. Participants appreciated the immersive experience and real-time biofeedback, which can improve engagement and recovery outcomes.
- **Barriers to Adoption**: Despite the positive outlook, several challenges were identified that could limit the widespread adoption of VR haptics training.
  - Cost: The high price of VR and haptic equipment was seen as a significant barrier, especially for patients reliant on public healthcare services or without the means to afford such technology.
  - Accessibility: Participants expressed concerns about whether the technology would be made widely available through public health systems like the NHS or if it would remain a costly private treatment.
  - Usability: Many participants voiced discomfort with VR systems, with some experiencing motion sickness, particularly when their FND symptoms were more intense. Additionally, concerns were raised about the learning curve for patients unfamiliar with VR technology, and whether sufficient technical support would be available to assist them.
  - Overstimulation: A considerable portion of participants reported sensory overload while using VR, which could hinder its adoption, especially for those with heightened sensitivity due to FND.

Rehab Technologies Network
Loughborough University
University of Nottingham UK | CHINA | MALAYSIA
NRC
NHS Nottingham University Hospitals NHS Trust
Funded by
UKRI Engineering and Physical Sciences Research Council

U.S. FDA GUIDELINES FOR DELPHI ROUND 2

NRC Rehabilitation Technologies Conference

FDA U.S. FOOD & DRUG ADMINISTRATION

Augmented Reality and Virtual Reality Medical Devices: Questions to Consider

- Is there clinical evidence for using XR (Extended Reality) in certain FND care?
- Are there benefits to using XR in certain FND cases?
- Are there limitations to who can use XR in FND?
- What training and education are needed to safely and effectively use XR in FND?
- How will XR change a FND care pathway?
- How do patients transition to alternative treatment techniques when needed?
- How can patients be sure XR is helping?
- Does XR pose any physical risks to healthcare professionals?
- What are the risks to FND patients?

Rehab Technologies Network
Loughborough University
University of Nottingham UK | CHINA | MALAYSIA
NRC
NHS Nottingham University Hospitals NHS Trust
Funded by UKRI Engineering and Physical Sciences Research Council

COST-EFFECTIVENESS ANALYSIS

- **One-Size-Fits-All Approach**: Traditional CEA uses a standardized formula to calculate QALY, which does not account for variations in patient conditions. It treats health improvements as equally valuable across different patients, regardless of their baseline quality of life (QoL).
- **Quality of Life (QoL) Bias**: Sicker or more disabled patients have a lower starting quality of life score. Therefore, any improvements in their condition are inherently valued as less in the QALY formula, even if the improvement is substantial for them.
- **Unequal Valuation of Health Gains**: The calculation values a 0.1 improvement in QoL equally for all patients. However, a 0.1 gain in QoL for a severely ill patient (e.g., someone with Alzheimer's) may be far more impactful than the same 0.1 gain for a relatively healthy patient (e.g., someone with acid reflux). The current model does not capture this nuance, leading to a disparity in how different patient populations are assessed.
- **Limitations in Addressing Disability**: The model implicitly devalues the lives of more disabled or sicker individuals by assigning lower weights to their health improvements, potentially leading to inequitable decisions about which treatments are deemed cost-effective.

UNDERSTANDING OF COST-EFFECTIVENESS ANALYSIS FROM PATIENT-CENTRIC VALUE ASSESSMENT – DELPHI ROUND 2

NRC Rehabilitation Technologies Conference

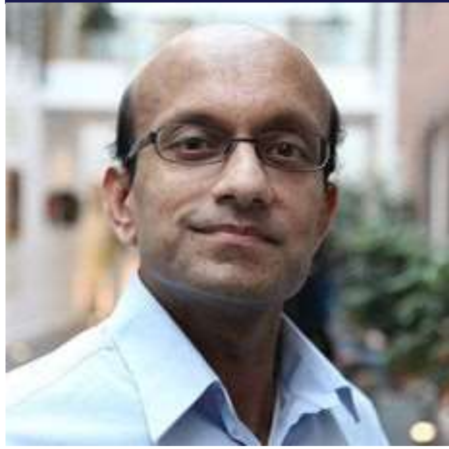

Prof. Siddhartha Bandyopadhyay

- **Ask two key questions**:
  - What is untreated quality of life (QoL)? It considers how severe a patient's condition is without any intervention.
  - How much would value improvements in quality of life and additions to life expectancy? It factors in how a FND patient personally values health gains.
- **Four ways to adjust traditional cost-effectiveness models**:
  - Disease Severity: FND patients with severe illnesses are willing to invest more for small improvements in health compared to those with milder conditions.
  - Disability: FND patients with permanent disabilities are willing to pay more for health improvements, as their baseline QoL is lower.
  - Added Health Benefits: Health improvements for sicker patients are considered to provide more value than the same improvements for healthier individuals.
  - Uncertainty: XR treatments with high risks of failure are valued less by patients who are risk-averse, meaning that uncertainty plays a role in patient preferences.

Rehab Technologies Network | Loughborough University | University of Nottingham UK | CHINA | MALAYSIA | NRC | NHS Nottingham University Hospitals NHS Trust | Funded by UKRI Engineering and Physical Sciences Research Council

SUMMARY

Barriers to Adoption

Next Steps

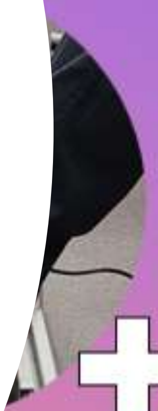

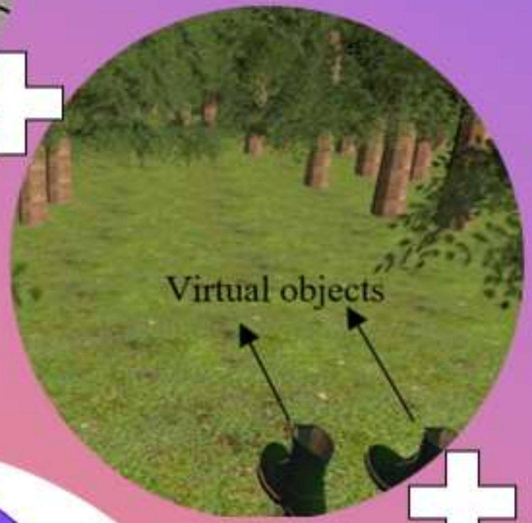

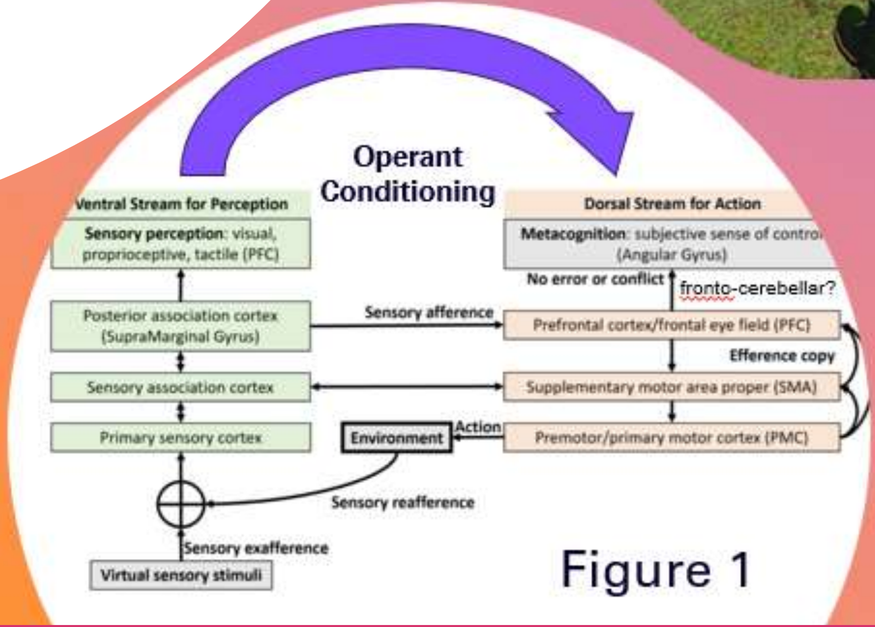

Figure 1

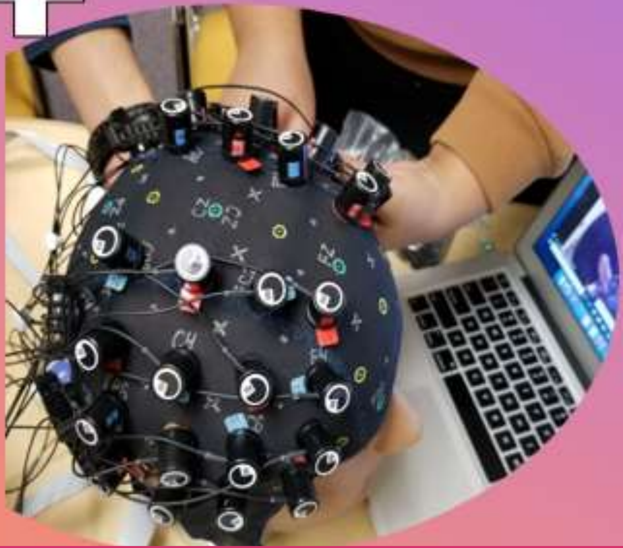

Summary

Barriers to Adoption

- **Cost**: Cost-effectiveness analysis with equity considerations for VR and haptic equipment, especially for patients reliant on public healthcare or with limited financial means.
- **Accessibility**: VR technology more widely available as consumer products leveraging the gaming industry to ensure it doesn't remain a costly.
- **Usability**: Minimize cybersickness associated with VR systems with augmented reality, particularly for patients with more intense FND symptoms. Co-create comprehensive training and ensure sufficient technical support for patients unfamiliar with VR technology to ease the learning curve.
- **Overstimulation**: Develop strategies to reduce sensory overload, especially for patients with heightened sensitivity due to FND, to facilitate the broader adoption of VR technology.

Delphi Round 2

Next round of the Delphi survey will focus on refining design requirements for the VR haptics system based on feedback from same participants.

- FDA's Augmented Reality and Virtual Reality Medical Devices: Questions to Consider
  - Topics of discussion will include the clinical evidence supporting AR/VR use, accessibility concerns, potential benefits for training or treatment, risks like sensory overload or motion sickness, and ethical considerations around device affordability and patient data privacy.
- Cost-effectiveness Analysis From Patient-centric Value Assessment
  - Discussions would focus on how models can adjust for disease severity, disability, added health benefits, and FND patient preferences. The goal would be to develop a framework that more accurately reflects the values and needs of vulnerable FND patients, which traditional CEA models often overlook.

Funded by

TEAM

CO-PRODUCTION OF A PLATFORM TECHNOLOGY FOR VR BIOFEEDBACK TRAINING UNDER OPERANT CONDITIONING FOR FUNCTIONAL LOWER LIMB WEAKNESS

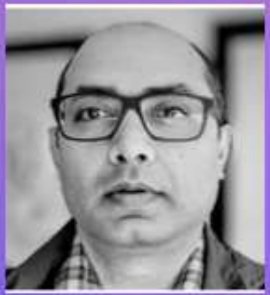
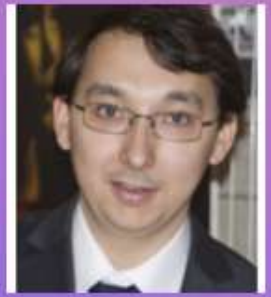
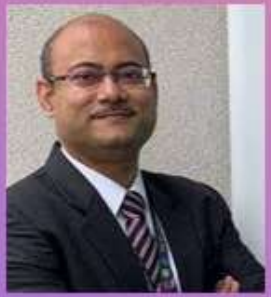
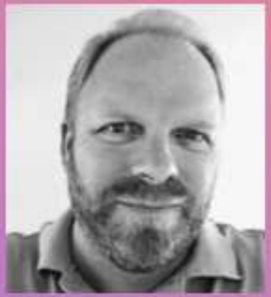
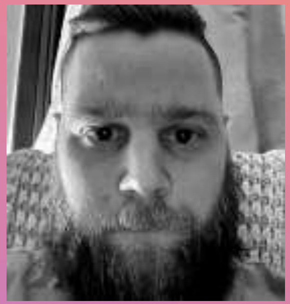

Anirban Dutta
University of Birmingham

Ildar Farkhatdinov
Kings College London

Abhijit Das
Lancashire Teaching Hospitals NHS Foundation Trust

Alastair Buchanan
Nudge Reality Pvt. Ltd.

Matthew Newsham
Patient Advocate

Katerina Hatjipanagioti
Patient Advocate

THANK YOU
Q&A

Rehab Technologies Network
Loughborough University
University of Nottingham UK | CHINA | MALAYSIA
NRC
NHS Nottingham University Hospitals NHS Trust
Funded by
UKRI Engineering and Physical Sciences Research Council
