# Supplementary material for: Extended Reality Biofeedback for Functional Upper Limb Weakness: Mixed Methods Usability Evaluation
Source: JMIR XR Spat Comput. 2025 Jun 3;2:e68580. doi: 10.2196/68580 (PMC12671321; doi:10.2196/68580)
Supplement: Multimedia Appendix 3 [file xr-v2-e68580-s003.pdf]

Portable Hi5 robot for neuromechanics investigation

This robotic interface is dedicated to investigating upper limb neuromechanics that span off from our clinical, neuroscience and human-robot interaction research*. The current design allows for controlled actuation of wrist or elbow flexion/extension. The compact and light portable interface can be quickly installed by mounting it to a table with clamps. Ergonomic wrist design and adjustable arm support ensure human-robot joint alignment providing comfortable natural movements for various sizes of hand/arm. It can be used for single hand actuation (replaceable left/right wrist handle) or for bi-manual or for human-human collaborative experiments. Torque and angular position sensing can be used for real-time feedback and to analyse performance and model individual neuromechanics. Maxon Motors driving technology is used for actuating wrist or elbow joints. The robot can be controlled in current, velocity and position control modes and programmed in Matlab, Matlab Simulink, and C/C++. The system can be easily used together with EMG and EEG sensing.

Key features:

- programmable torque/velocity/position
- gearless direct drive
- ergonomic wrist handle
- aligned human-robot joints
- optical encoder
- torque sensor
- portable and easy to install
- left/right replaceable wrist
- optional bi-manual configuration
- Matlab- and C- API code

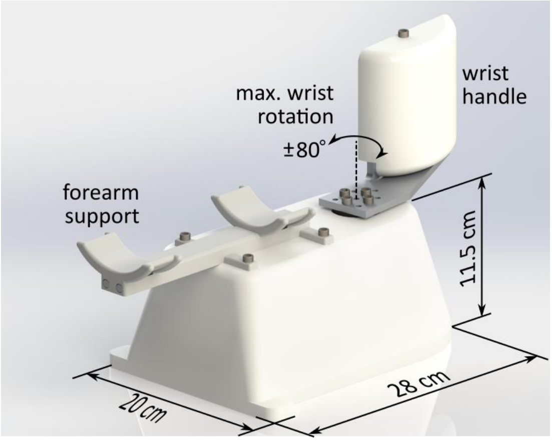

**Nominal torque**	1.4 Nm
**Nominal voltage**	30 V
**Nominal current**	5.95 A
**Peak torque**	13 Nm
**Peak current**	57 A
**Power**	240-800 W
**Range of rotation**	±80°
**Encoder resolution**	6000 CPT
**Power supply input**	115/230 VAC, 9 A
**Power supply output**	20-30 V, 26 A
**Total weight**	2 kg

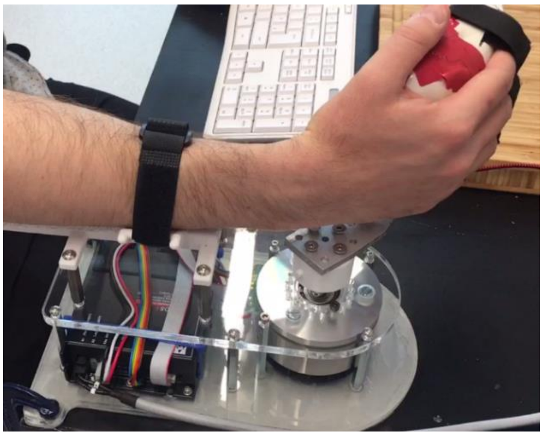

*VM McClelland et al. (2021), EEG measures of sensorimotor processing and their development are abnormal in children with isolated dystonia and dystonic cerebral palsy. NeuroImage 30: 102569.

DF Sakellariou et al. (2020), Abnormal microscale neuronal connectivity triggered by a proprioceptive stimulus in dystonia. Scientific Reports 10(1): 1-12.

A Takagi (2020), The dominant limb preferentially stabilizes posture in a bimanual task with physical coupling. Journal of Neurophysiology 123(6): 2154-60.

Y Li et al. (2020), Improving tracking through human-robot sensory augmentation. IEEE Robotics and Automation Letters 5(3).

E Ivanova et al. (2020), For motion assistance humans prefer to rely on a robot rather than on an unpredictable human. IEEE Open Journal of Engineering in Medicine and Biology 1: 133-9.

Y Li et al. (2019), Differential game theory for versatile physical human-robot interaction. Nature Machine Intelligence 1(1).

A Takagi et al. (2019), Haptic communication between humans is tuned by the hard or soft mechanics of interaction. PLoS Computational Biology 14(3): e1005971.

A Takagi et al. (2016), Motion plan changes predictably in dyadic reaching. PLoS One 11(12): e0167314.

A Takagi et al. (2016), Facing the partner influences exchanges in force. Scientific Reports 6(1): 1-6.

A Melendez-Calderon et al. (2015), Interpersonal strategies for disturbance attenuation during a rhythmic joint motor action. Physiology and Behavior 1(147): 348-58.
