# Supplementary material for: Extended Reality Biofeedback for Functional Upper Limb Weakness: Mixed Methods Usability Evaluation
Source: JMIR XR Spat Comput. 2025 Jun 3;2:e68580. doi: 10.2196/68580 (PMC12671321; doi:10.2196/68580)
Supplement: Multimedia Appendix 4 [file xr-v2-e68580-s004.pdf]

RehabTech+ XR Game Concepts

© Copyright Nudge Reality Ltd 2024

Introduction

This document introduces a novel immersive rehabilitation application designed specifically for patients with Functional Neurological Disorder (FND) who experience limited arm movement. Utilising a cutting-edge robotic arm, this application provides haptic feedback and variable resistance within a single horizontal plane. This mechanism progressively supports users, reducing assistance as they gain strength and control over their movements.

To enhance the rehabilitation experience, we have developed three engaging game concepts: **Hoop Hustle**, **Ring Master**, and **Twist'n'Ring**. Each game is designed to motivate and challenge patients, aiding in their physical therapy through fun and interactive gameplay.

Game Concepts

1. Hoop Hustle

"Hoop Hustle" is a reaction-based game that tests precision, timing, and skill. Players twist their wrist to control a basketball hoop along an arc beneath ball droppers, catching balls as they fall. The game's difficulty increases with speed and distance, enhancing reflexes and control.

2. Ring Master

In "Ring Master," players use a wrist-twisting motion to control a claw that places coloured rings on target poles. The game combines strategy and quick reactions, with progressive difficulty and special rings adding complexity. The goal is to match three or more rings of the same colour to score points.

3. Twist'n'Ring

"Twist'n'Ring" immerses players in a VR environment where they throw rings at targets using wrist twists. The game challenges players to master precision and technique, with progressive levels introducing dynamic obstacles and environmental factors.

These games not only make rehabilitation engaging but also provide a measurable way to track progress and improve arm movement in FND patients. Each game leverages realistic physics, intuitive controls, and vibrant visuals to create an immersive and therapeutic experience.

© Copyright Nudge Reality Ltd 2024

1. Game Pitch: Hoop Hustle

Welcome to "Hoop Hustle," an exhilarating reaction-based game where precision, timing, and skill are the keys to victory. Twist your wrist to control a basketball hoop on an arc beneath five different ball droppers. The challenge? Catch the balls in the hoop as they fall, but with a twist – the further you need to move the hoop, the harder it gets!

Gameplay Mechanics:

Intuitive Controls: Players use a motion-sensing controller to twist their wrist, moving the basketball hoop left and right along a curved arc beneath the ball droppers. The hoop's movement is sensitive to the speed and angle of your wrist twist, making for an immersive and responsive gameplay experience.

Progressive Difficulty: As players advance through levels, the balls will drop at varying speeds, requiring quicker reflexes and more precise control. The arc gets wider, and the hoop becomes more challenging to move as you progress, demanding greater effort and dexterity.

Dynamic Obstacles: Some levels might feature barriers that the hoop must break through with enough force, we could have special balls that grant extra points or slow down time temporarily. Power-ups can be collected to ease movement difficulty, increase hoop size, or slow down ball drops for a limited time.

Key Features:

Realistic Physics: Advanced physics engine ensures each ball drop and hoop movement feels authentic, mimicking real-life basketball dynamics.

Customizable Difficulty: Players can choose from various difficulty settings, from beginner-friendly to expert level, ensuring fun for all skill levels.

Engaging Visuals and Sounds: Crisp, vibrant graphics and realistic sound effects enhance the gaming experience, making every catch satisfying.

Multiple Modes:

Arcade Mode: Endless play for high scores.

Challenge Mode: Time-based challenges with escalating difficulty.

Training Mode: Practise your skills and master the controls.

© Copyright Nudge Reality Ltd 2024

2. Game Pitch: Ring Master

Enter the world of "Ring Master," a captivating reaction-based puzzle game where precision, timing, and strategy blend seamlessly. Use a wrist-twisting motion to control a claw that moves across an arc above five target poles. Drop coloured rings onto these poles to create matches of three or more same-coloured rings for a satisfying cascade of points. Need more rings? There's a dispenser pole at your disposal. But be prepared – the further you move the claw, the more effort it takes!

Gameplay Mechanics:

Intuitive Controls: Players use a motion-sensing controller to twist their wrist, moving the claw left and right along a curved arc above the target poles. The claw's movement is directly responsive to the player's wrist motion, offering a fluid and immersive control experience.

Progressive Difficulty: As players advance, the claw's movement arc widens, requiring more effort and precision to move it across to the poles. Ring drop speed increases, and new ring colours are introduced to raise the challenge.

Strategic Gameplay: Match three or more rings of the same colour on a pole to clear them and earn points. Chain multiple matches for combo bonuses. Use the dispenser pole to restock rings when you run out. Plan your moves to optimise ring placement and maximise points.

Dynamic Elements: Introduce special rings with unique effects, such as bombs that clear surrounding rings, or rainbow rings that match with any colour. Include obstacles and barriers that alter the ring's trajectory or temporarily block the claw's movement, adding a layer of strategy.

Realistic Physics: A sophisticated physics engine ensures that rings drop and stack naturally, providing a realistic and engaging gameplay experience.

Customizable Difficulty: Various difficulty settings accommodate players from beginners to experts, with adjustable speed and arc width.

Vibrant Visuals and Sound: Colourful graphics and immersive sound effects create a captivating gaming environment.

Multiple Modes:

Classic Mode: Endless play focused on achieving the highest score.

Challenge Mode: Timed challenges and specific goals to test your skills.

Puzzle Mode: Pre-set levels with unique layouts and ring combinations to solve.

© Copyright Nudge Reality Ltd 2024

3. Game Pitch: Twist'n'Ring

Welcome to "Twist'n'Ring" an innovative and immersive virtual reality game where players use a single controller to twist their wrist and throw rings varying distances to hit targets for different scores. This unique control scheme challenges players to master precision and technique in a series of engaging environments and gameplay modes.

Gameplay Mechanics:

Twist Wrist Controls: Players use a single controller and use wrist-twisting motions to control the direction, strength, and angle of each ring throw. The VR system accurately tracks the twist motion, translating it into realistic ring throws with natural physics. Players curl their wrist to stop a visible arc which shows the throwing direction, and then straighten their wrists with the rings being thrown based on the power used.

Scoring System: Targets are placed at various distances and angles, each with different point values based on difficulty. Targets can have concentric scoring zones, with higher points awarded for hitting closer to the centre. Players earn bonus points for consecutive hits, hitting multiple targets in quick succession, and achieving specific challenges.

Progressive Difficulty: Initial levels feature close, stationary targets, gradually introducing farther distances, moving targets, and obstacles. Environmental factors such as wind, floating platforms, and dynamic barriers add complexity as players advance.

Special Rings and Power-ups: Special rings with unique properties can be collected, such as rings that explode upon impact, rings that slow down time, or rings that expand their hit area. Power-ups and special rings are earned through gameplay achievements or found within levels.

Multiple Game Modes:

Classic Mode: Endless play with increasing difficulty, focusing on achieving the highest score possible.

Challenge Mode: Time-based and objective-specific challenges that test player skills in different scenarios.

Key Features:

Realistic Physics Engine: An advanced physics engine ensures that every ring throw behaves realistically, responding accurately to the player's wrist twists.

Instant Feedback System: Detailed feedback on throw strength, angle, and accuracy helps players refine their technique.

© Copyright Nudge Reality Ltd 2024
