# Supplementary material for: Extended Reality Biofeedback for Functional Upper Limb Weakness: Mixed Methods Usability Evaluation
Source: JMIR XR Spat Comput. 2025 Jun 3;2:e68580. doi: 10.2196/68580 (PMC12671321; doi:10.2196/68580)
Supplement: Multimedia Appendix 5 [file xr-v2-e68580-s005.pdf]

Usability Testing Script for XR Technology Platform

Introduction:

Hello, my name is __________, and I'll be guiding you through this session today.

Before we start, I want to go over some important details with you.

We're testing an XR (Extended Reality) platform designed to help manage Functional Neurological Disorders. This session should take about an hour. The main thing to remember is that we're testing the platform, not you, so there's no right or wrong way to interact with it. Your feedback will help us improve the system.

As you go through each task, please try to think out loud. Let us know what you're seeing, trying to do, and what you're thinking. Honest reactions and comments are welcome, and if you have any questions, feel free to ask.

If you're comfortable, I'd like you to sign a permission form that allows us to record this session for research purposes.

Do you have any questions so far?

Pre-Test Questions:

Can you tell me a bit about your background or occupation?

How comfortable are you with using XR or similar digital technologies?

Have you ever used XR technology for therapy or training before?

Platform Exploration:

OK, let's start with a quick exploration.

I'd like you to look around the XR platform interface. What's the first thing you notice?

Without interacting too much, can you describe what you think the purpose of this platform is?

Who do you think this platform is intended for?

What do you think you can do with this platform?

Tasks:

Now, I'll give you some specific tasks to try. Remember to think out loud as you go through them.

Task 1: VR Relaxation Session

Objective: To assess relaxation features in a virtual environment.

Instructions: Start a VR relaxation session and navigate through the setup. How easy or difficult is it to set up and enter relaxation mode?

Follow-up: How was that experience? What worked well, and what didn't?

Task 2: XR Position Feedback Control

Objective: To test motor control feedback in XR.

Instructions: Using the haptic feedback feature, perform a guided movement task. Follow the on-screen or auditory instructions.

Follow-up: How did you find the haptic feedback? Was the guidance clear, and did it feel natural?

Task 3: XR Force Feedback Control

Objective: To evaluate usability of force feedback in controlling movements.

Instructions: Try a force feedback control task, adjusting settings as needed. Perform movements and describe the sensations.

Follow-up: How easy was it to adjust the settings? Was the feedback helpful, or did you encounter any challenges?

Post-Task Rating:

After each task, please rate your experience on a scale of 1 to 5 (1 = Not Difficult, 5 = Extremely Difficult).

Task 1: VR Relaxation Session

Rating: _____

What made the task {insert participant's rating}?

Task 2: XR Position Feedback Control

Rating: _____

What made the task {insert participant's rating}?

Task 3: XR Force Feedback Control

Rating: _____

What made the task {insert participant's rating}?

Debriefing Questions:

What are your overall thoughts on the XR platform?

Which feature did you find most useful or enjoyable?

What did you like the least, or what caused the most difficulty?

Do you have any suggestions to improve the platform, especially for individuals with Functional Neurological Disorders?

Conclusion:

Thank you very much for your time and feedback today. Your insights are incredibly valuable in helping us improve this XR platform for FND therapy.
