# Supplementary figures and images for: Extended Reality Biofeedback for Functional Upper Limb Weakness: Mixed Methods Usability Evaluation
Source: JMIR XR Spat Comput. 2025 Jun 3;2:e68580. doi: 10.2196/68580 (PMC12671321; doi:10.2196/68580)

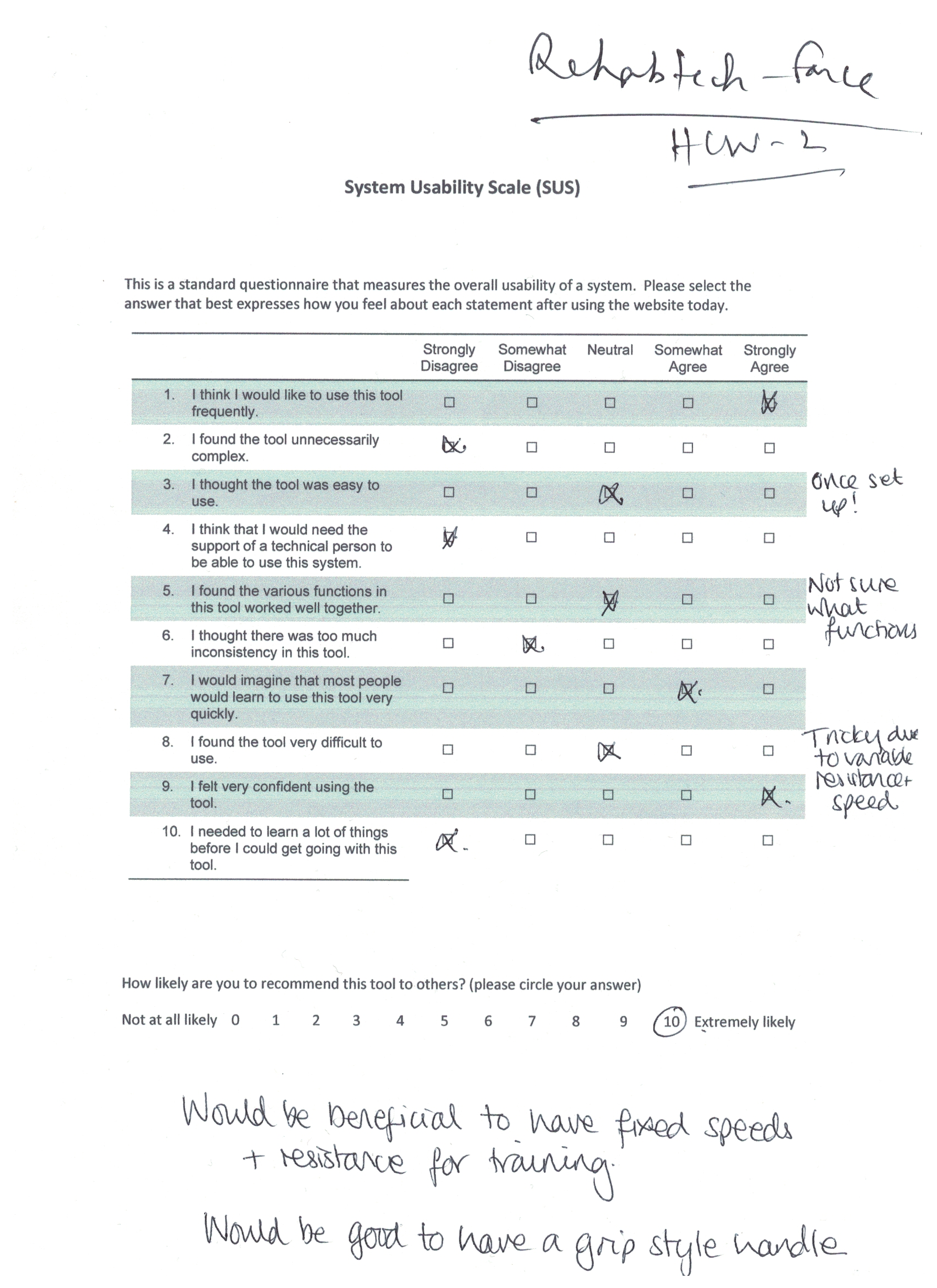

Supplement: Multimedia Appendix 6 [file xr-v2-e68580-s006.zip › ForceFeedback/Scan_1.png]

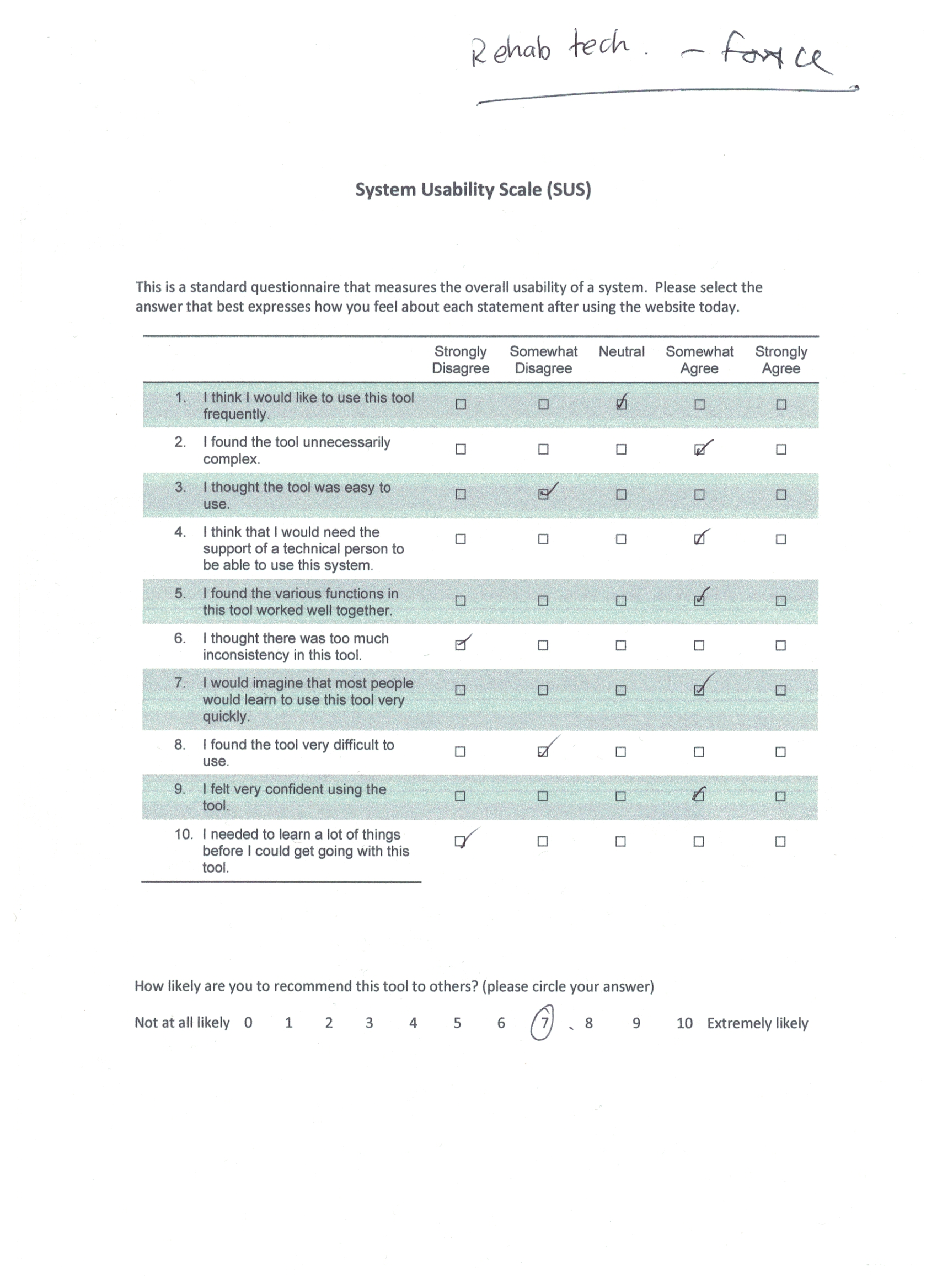

Supplement: Multimedia Appendix 6 [file xr-v2-e68580-s006.zip › ForceFeedback/Scan_2.png]

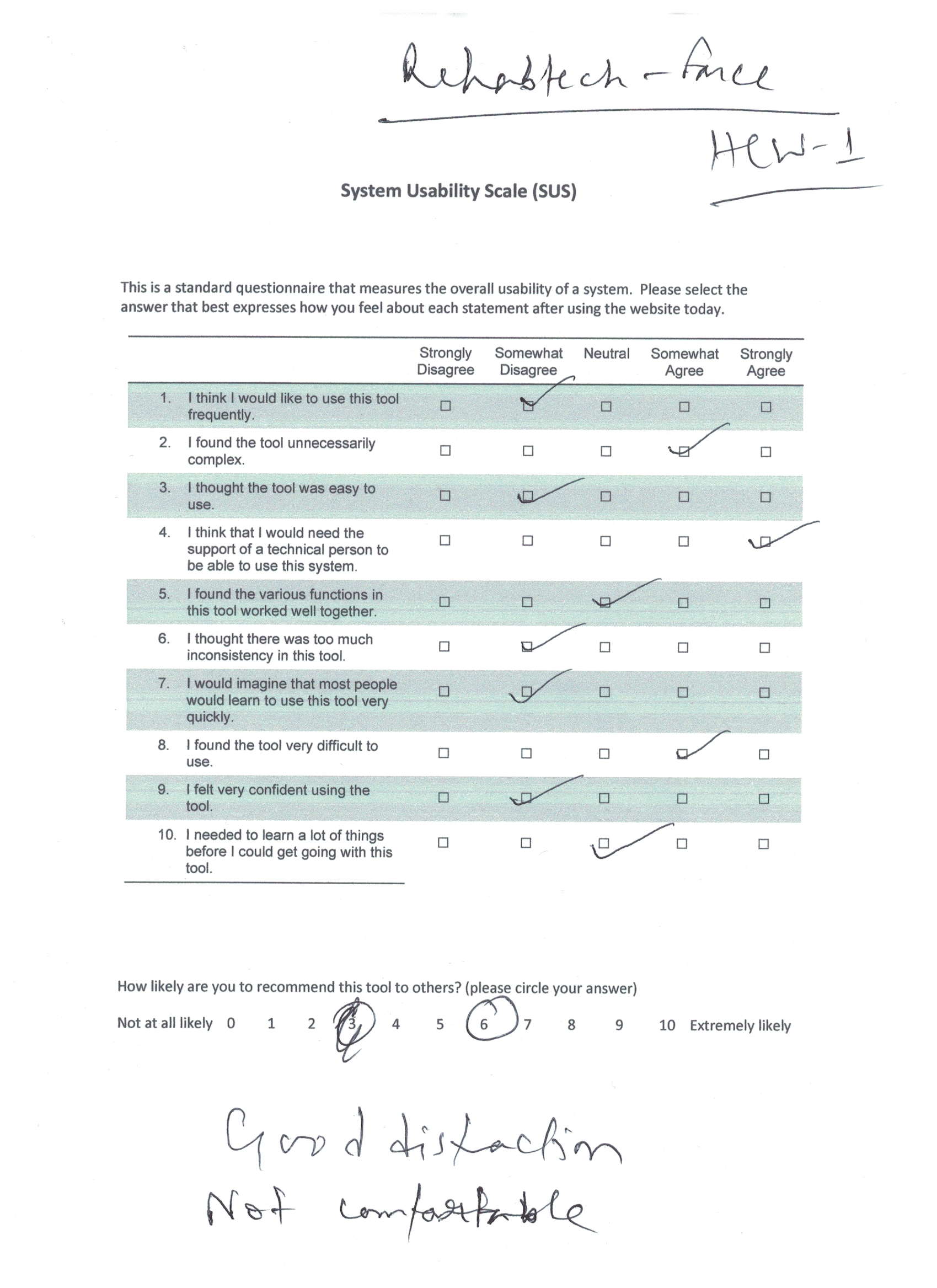

Supplement: Multimedia Appendix 6 [file xr-v2-e68580-s006.zip › ForceFeedback/Scan_3.png]

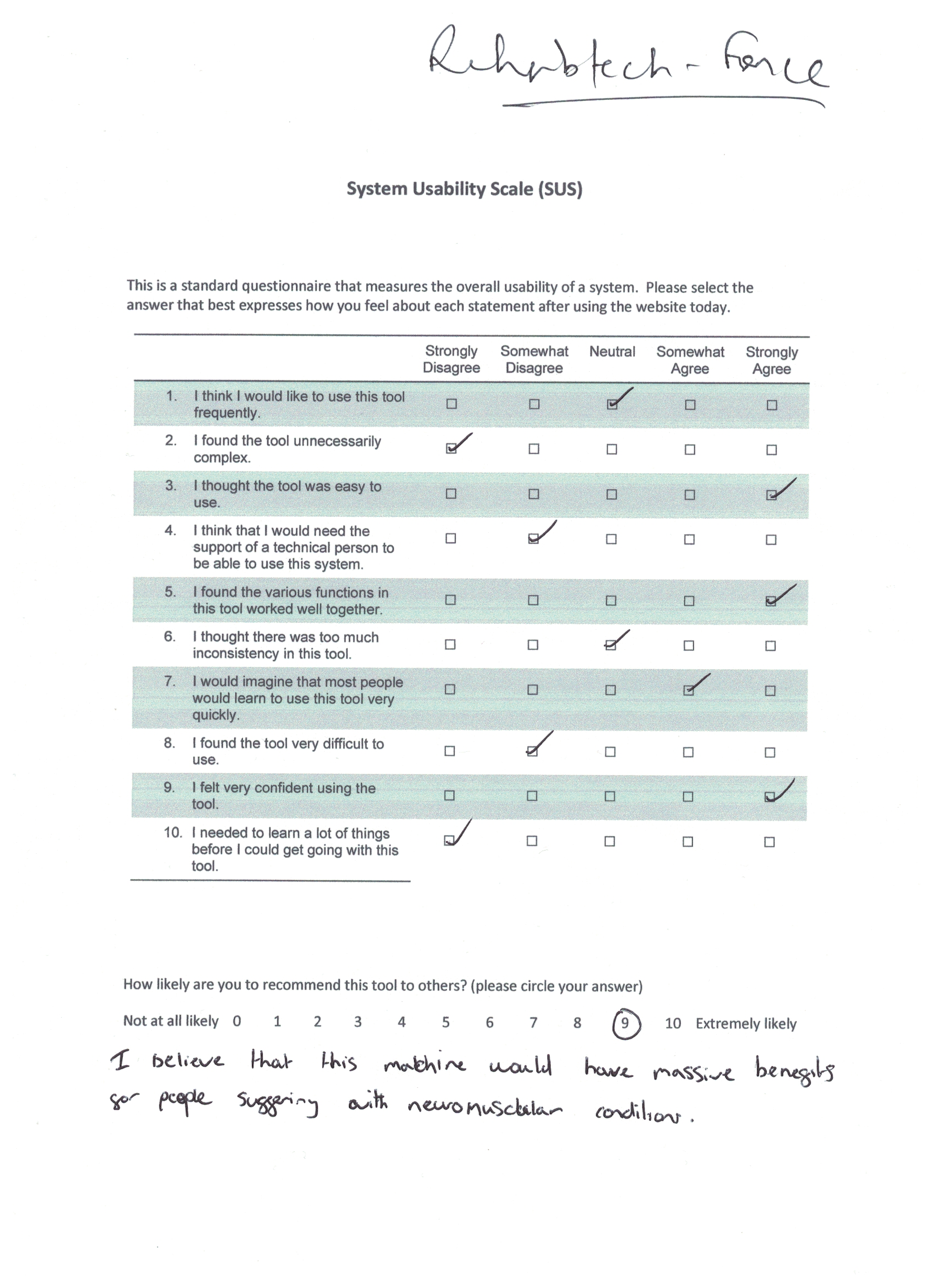

Supplement: Multimedia Appendix 6 [file xr-v2-e68580-s006.zip › ForceFeedback/Scan_4.png]

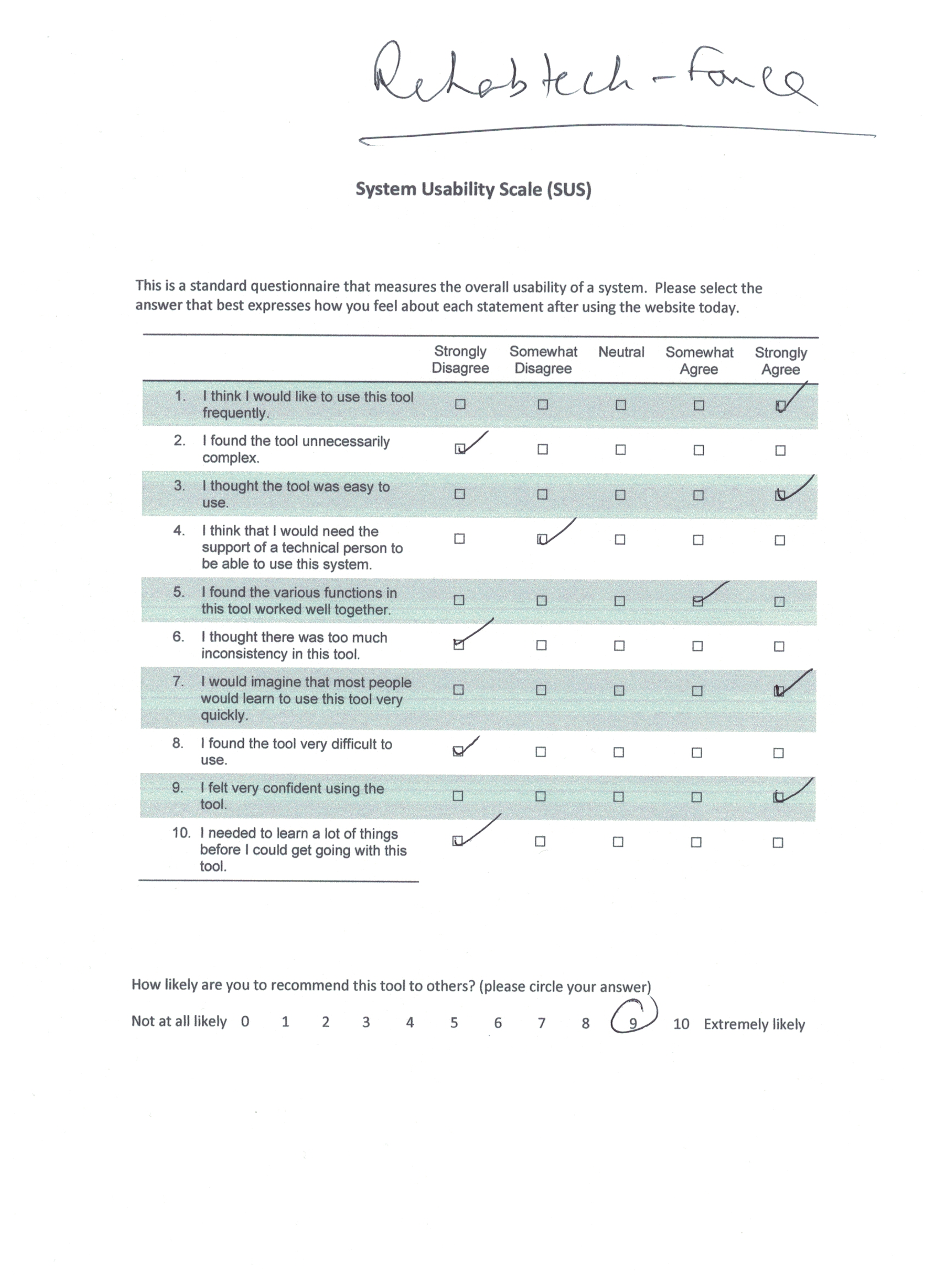

Supplement: Multimedia Appendix 6 [file xr-v2-e68580-s006.zip › ForceFeedback/Scan_5.png]

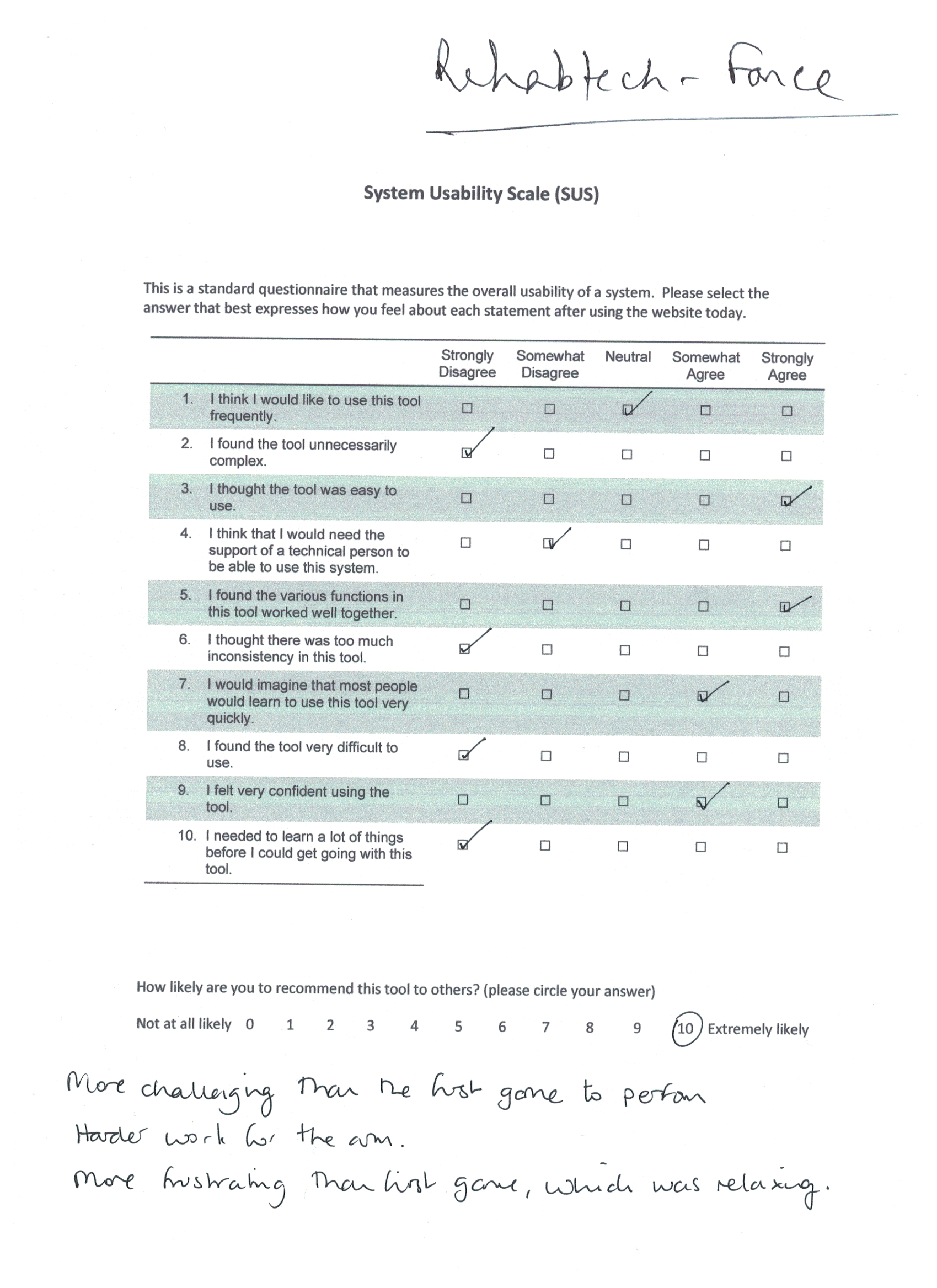

Supplement: Multimedia Appendix 6 [file xr-v2-e68580-s006.zip › ForceFeedback/Scan_6.png]

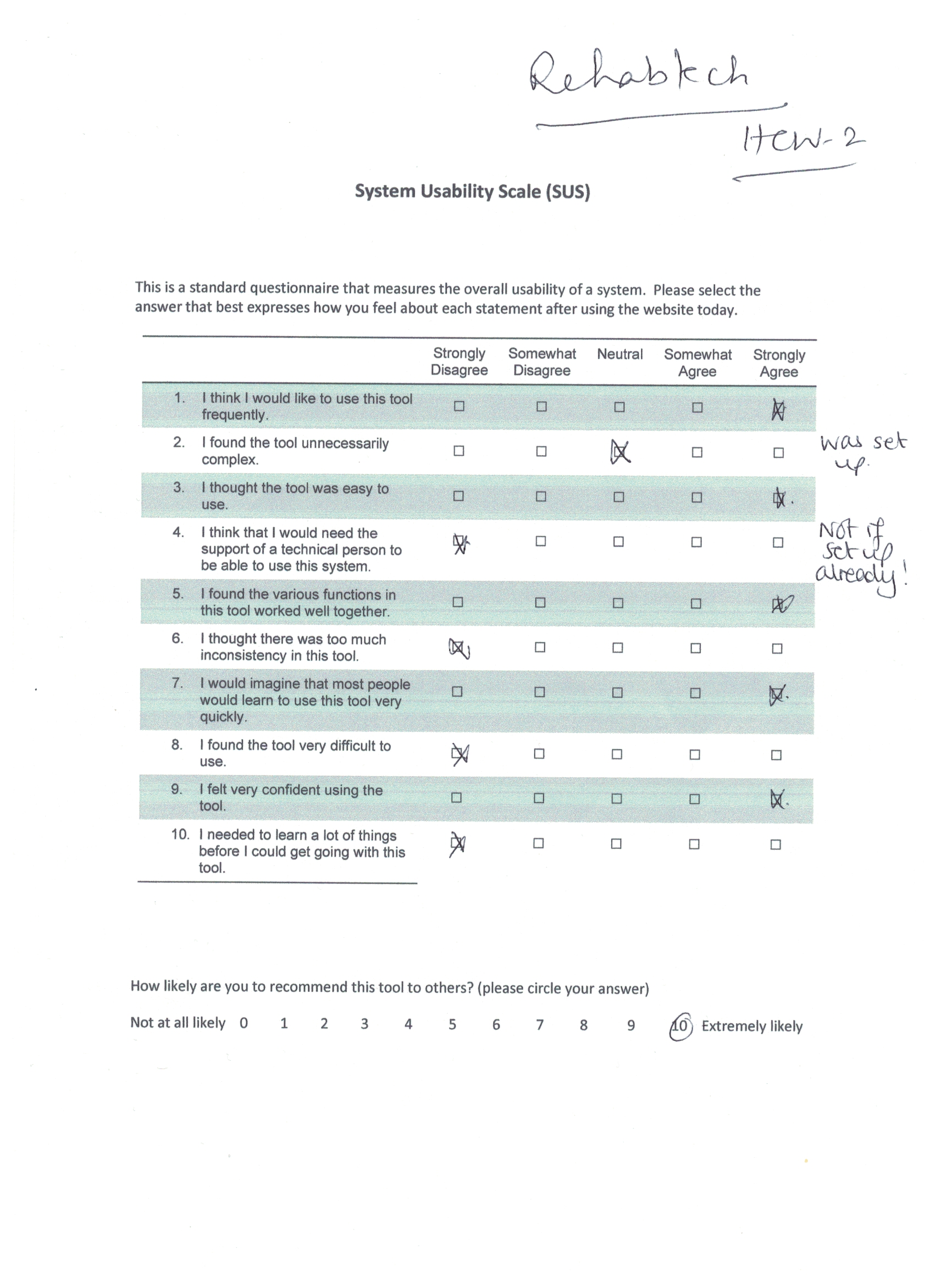

Supplement: Multimedia Appendix 7 [file xr-v2-e68580-s007.zip › PositionFeedback/Scan_1.png]

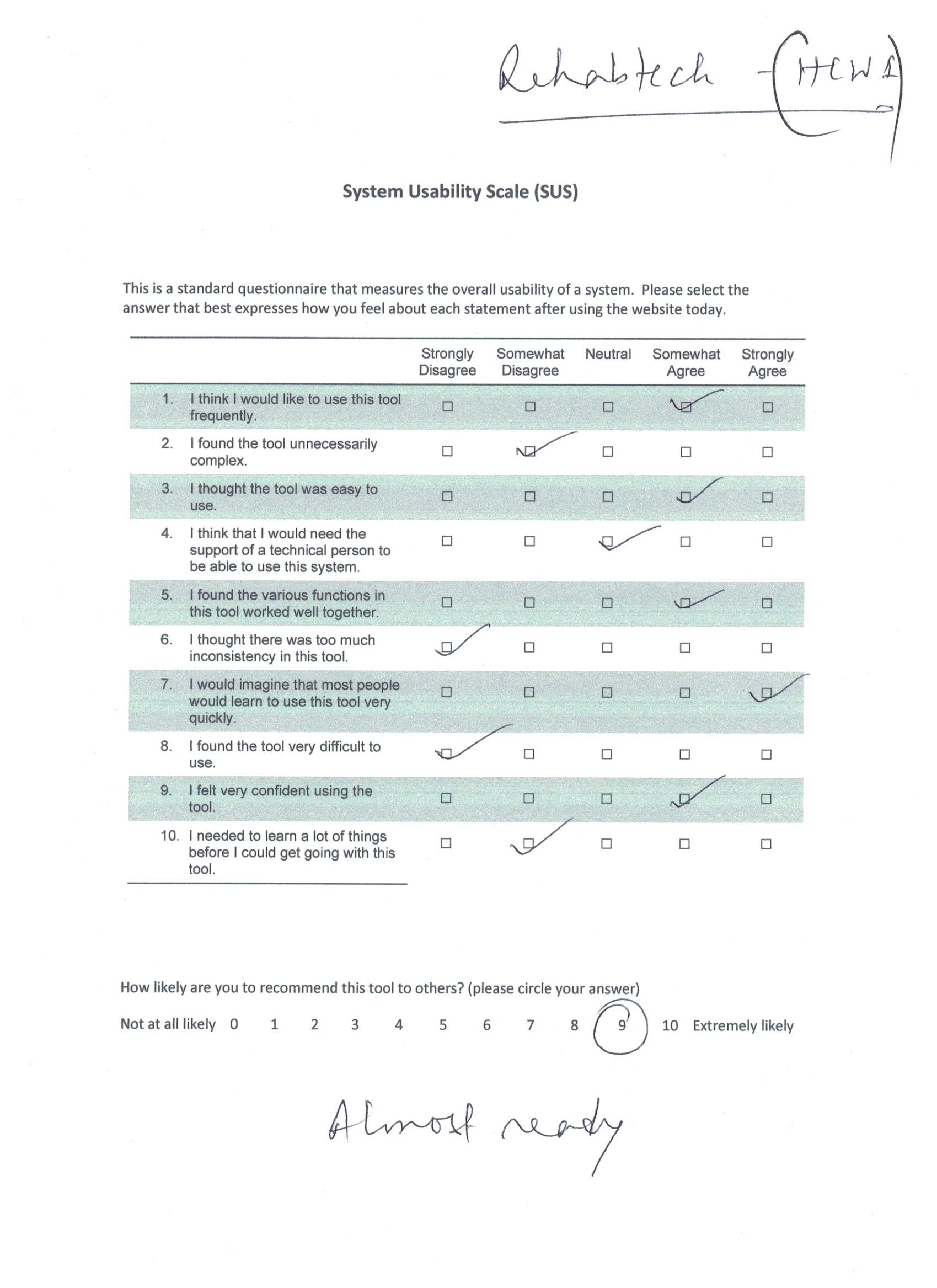

Supplement: Multimedia Appendix 7 [file xr-v2-e68580-s007.zip › PositionFeedback/Scan_2.png]

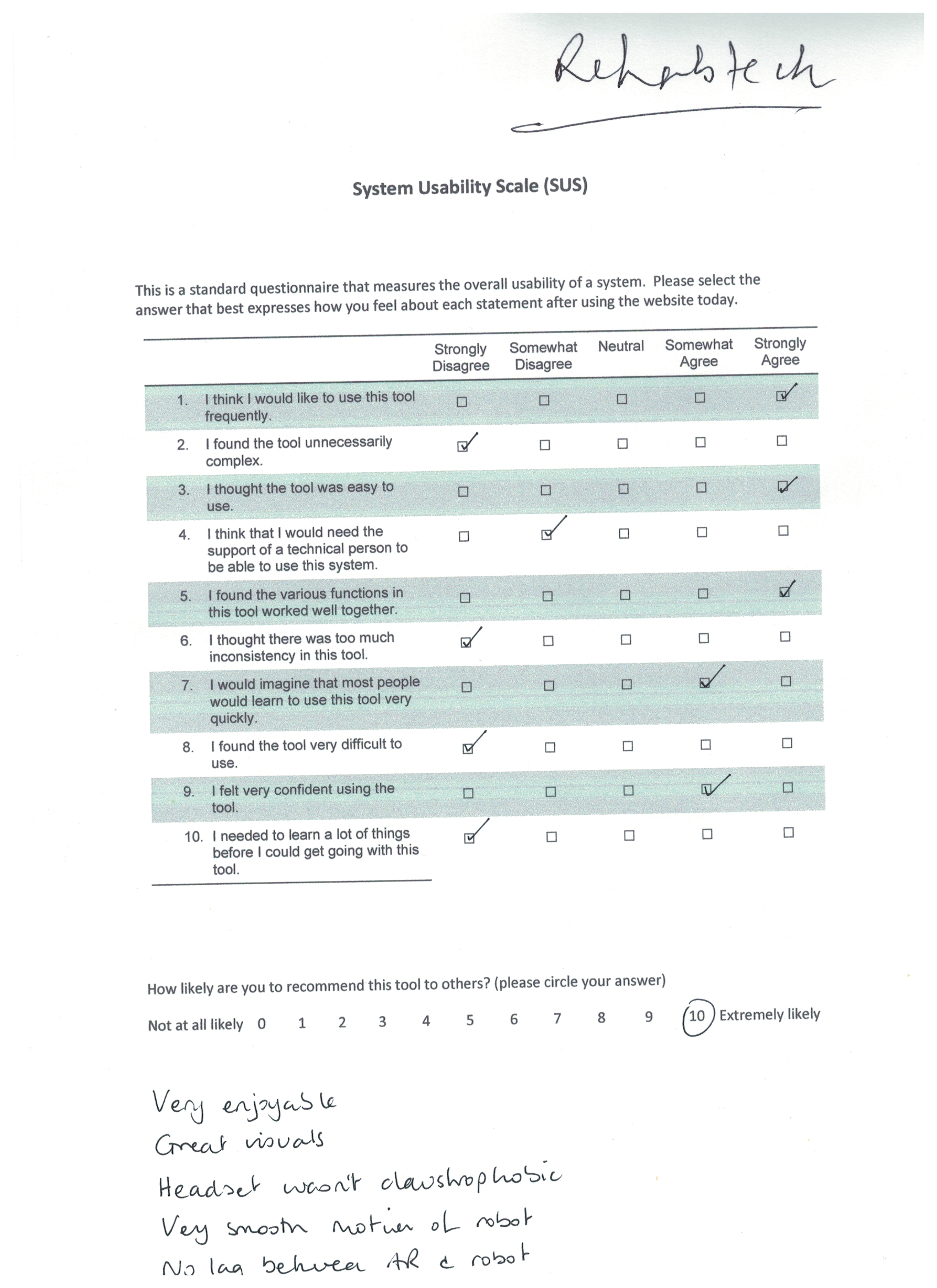

Supplement: Multimedia Appendix 7 [file xr-v2-e68580-s007.zip › PositionFeedback/Scan_3.png]

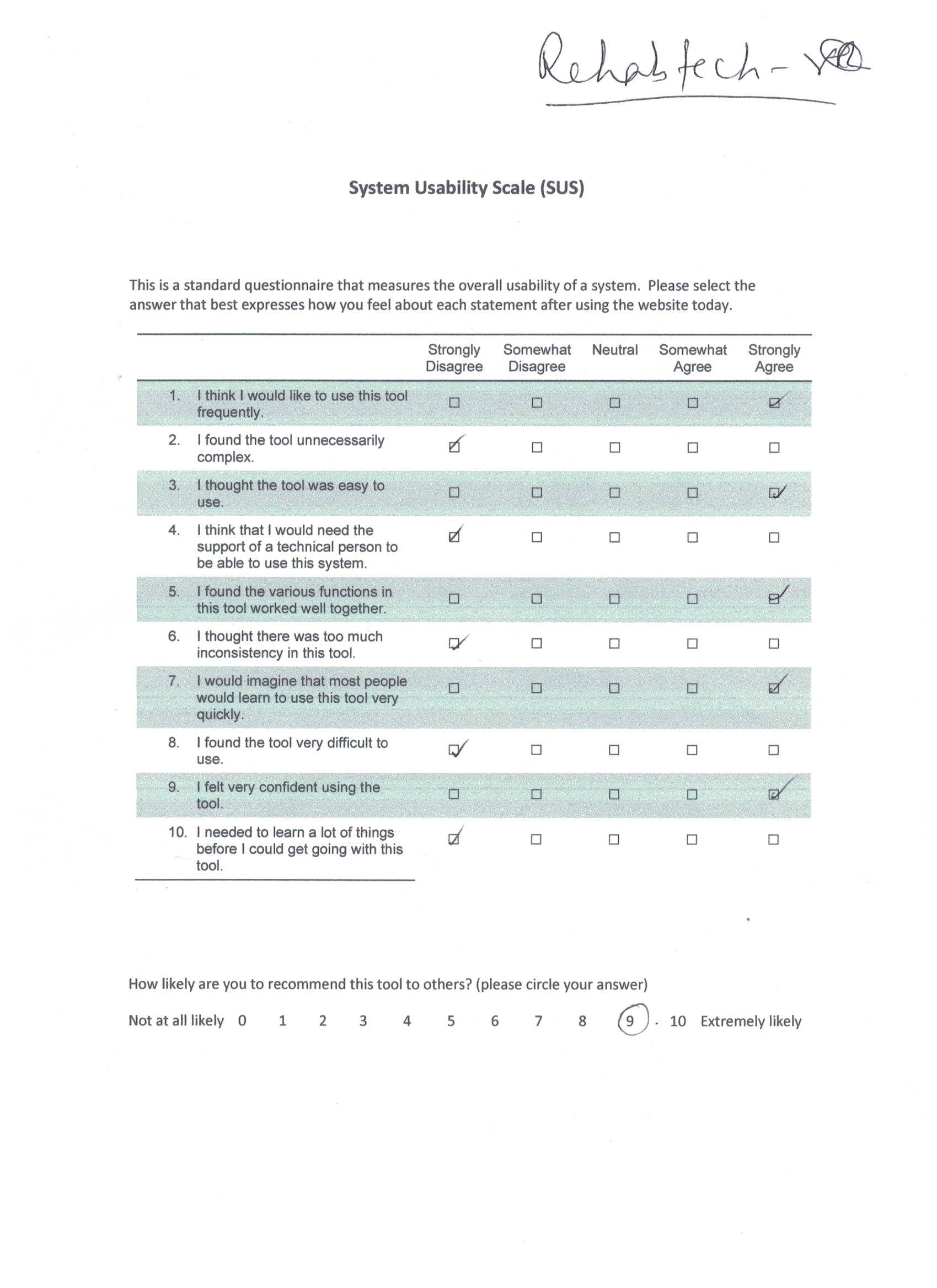

Supplement: Multimedia Appendix 7 [file xr-v2-e68580-s007.zip › PositionFeedback/Scan_4.png]

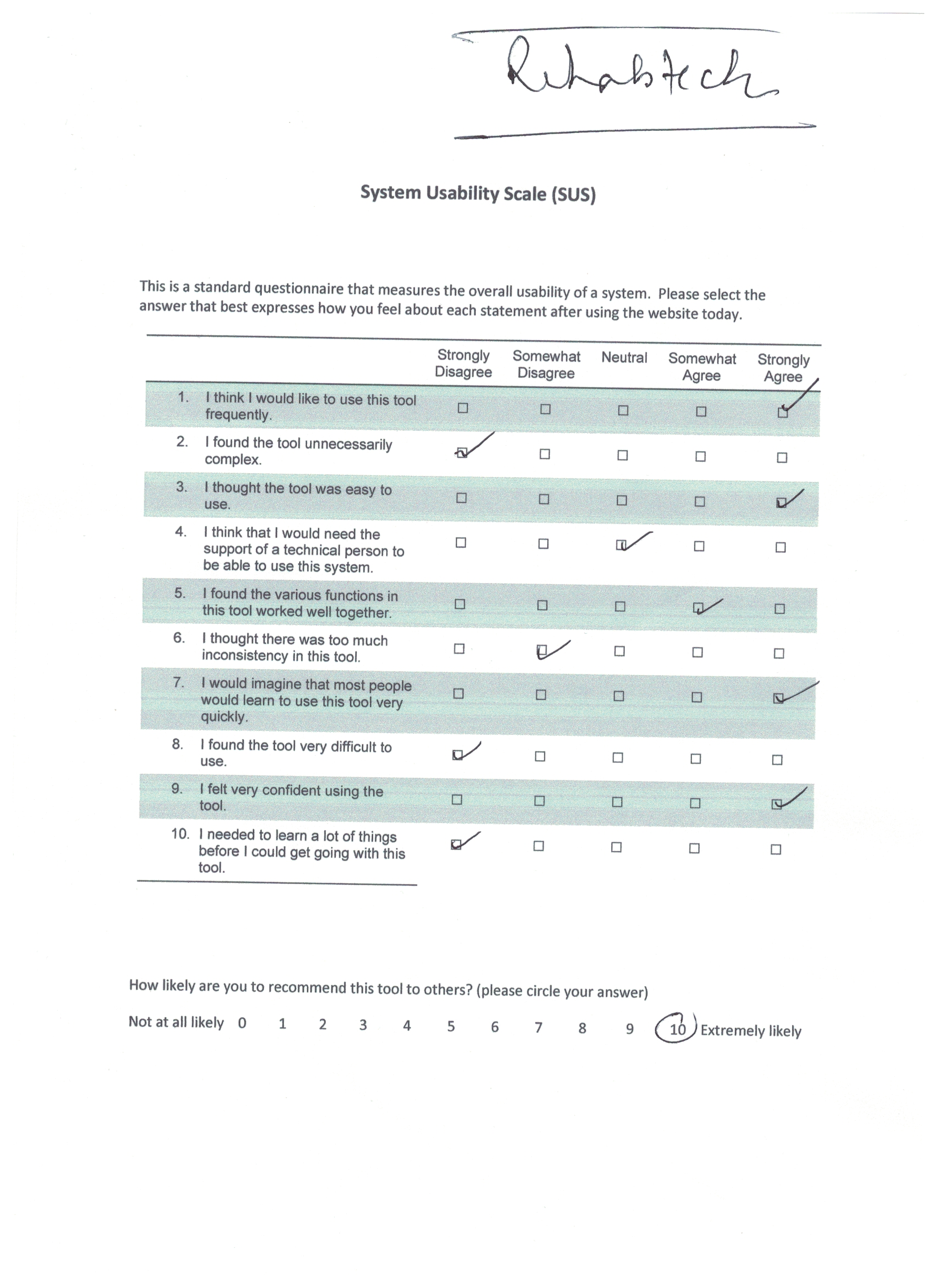

Supplement: Multimedia Appendix 7 [file xr-v2-e68580-s007.zip › PositionFeedback/Scan_5.png]

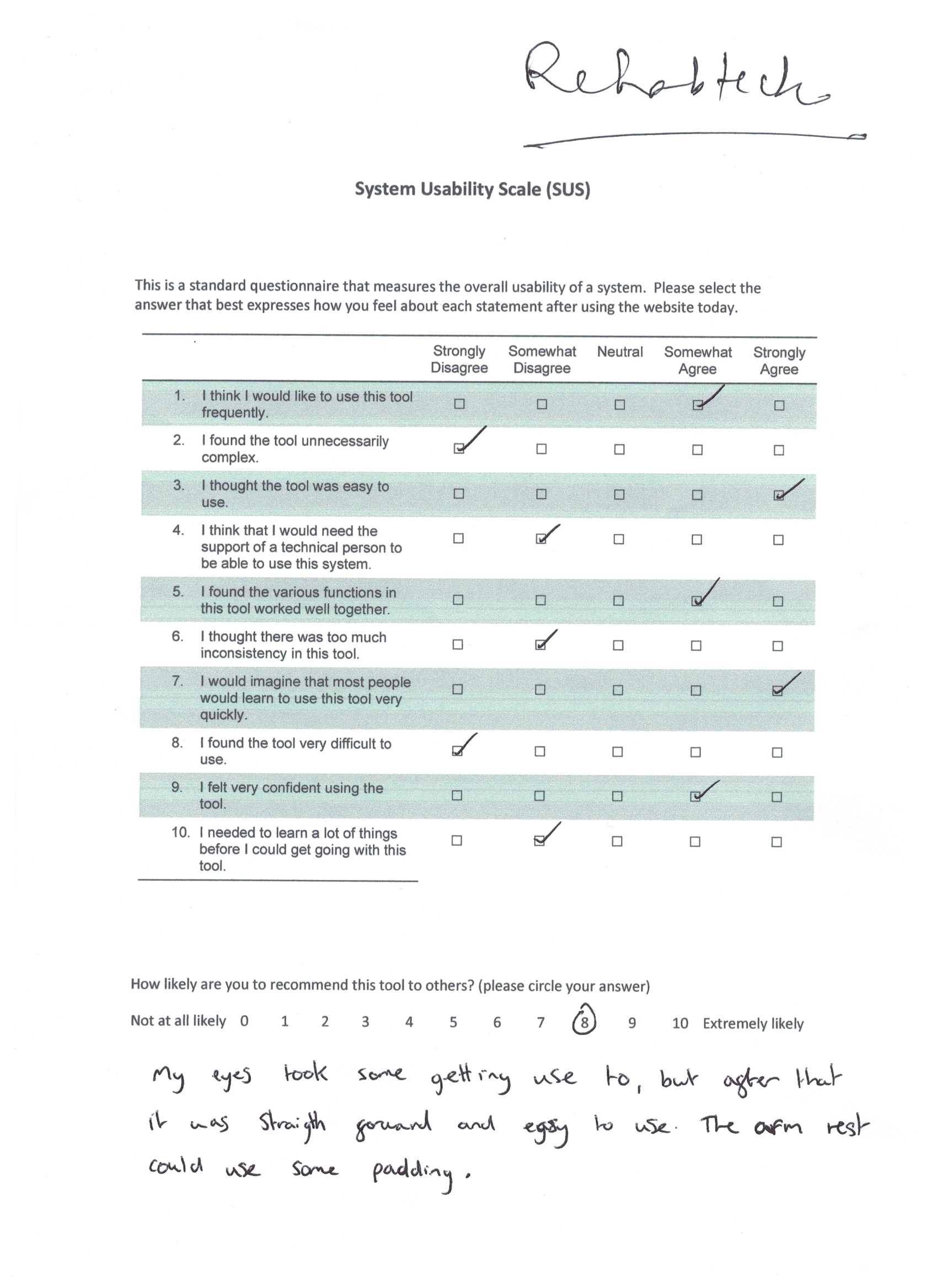

Supplement: Multimedia Appendix 7 [file xr-v2-e68580-s007.zip › PositionFeedback/Scan_6.png]

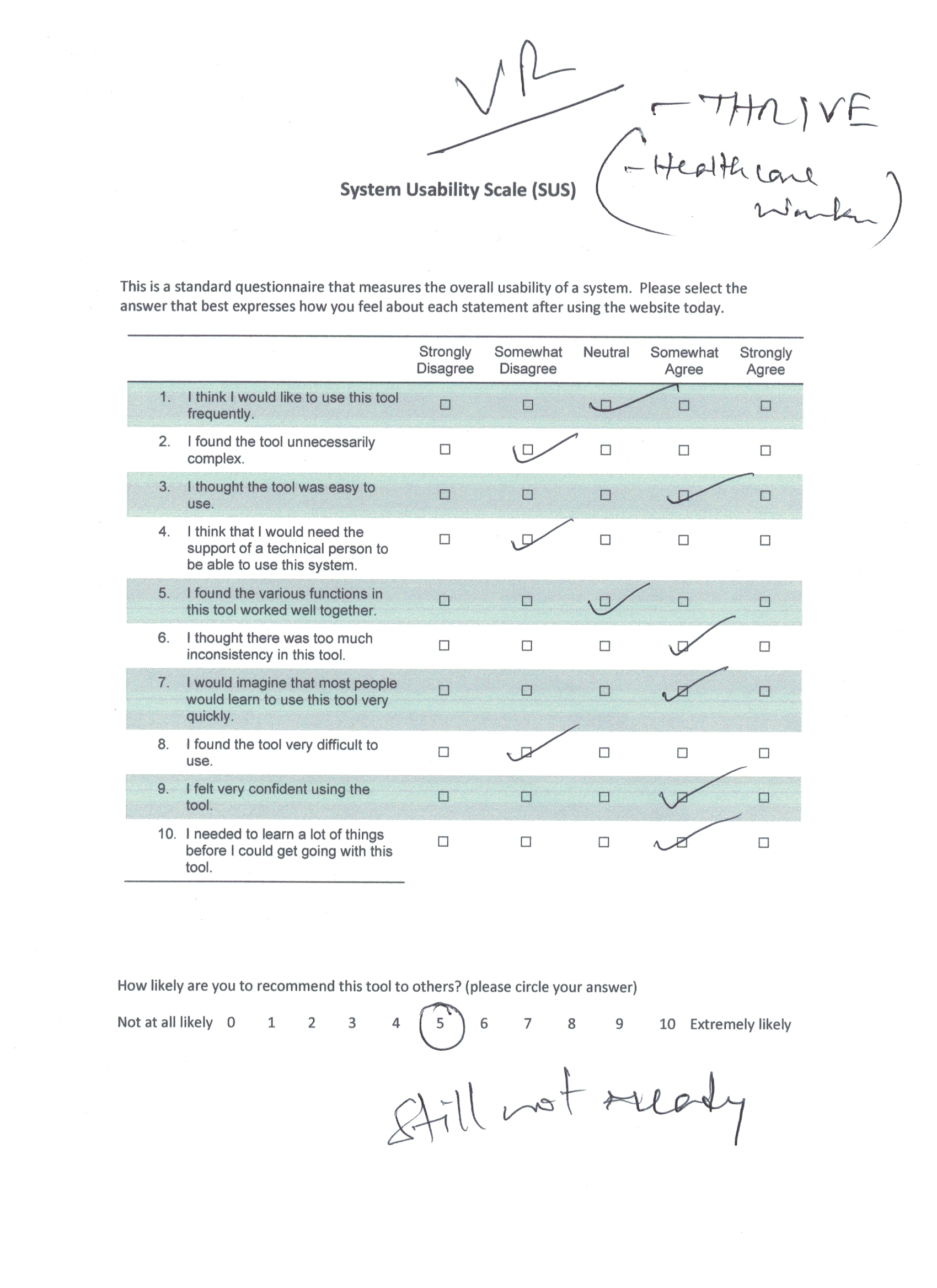

Supplement: Multimedia Appendix 8 [file xr-v2-e68580-s008.zip › Relaxation/Scan_1.png]

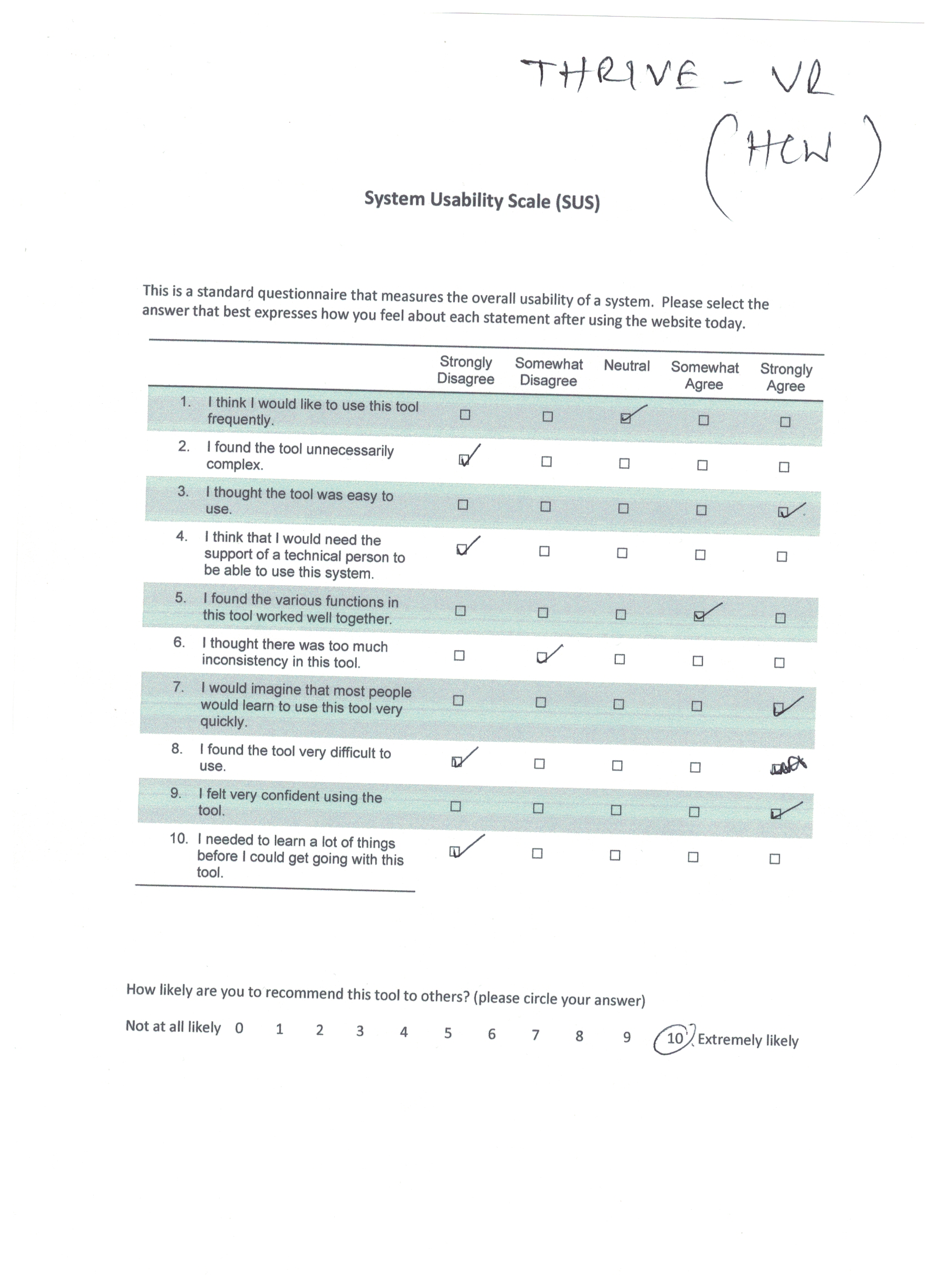

Supplement: Multimedia Appendix 8 [file xr-v2-e68580-s008.zip › Relaxation/Scan_2.png]

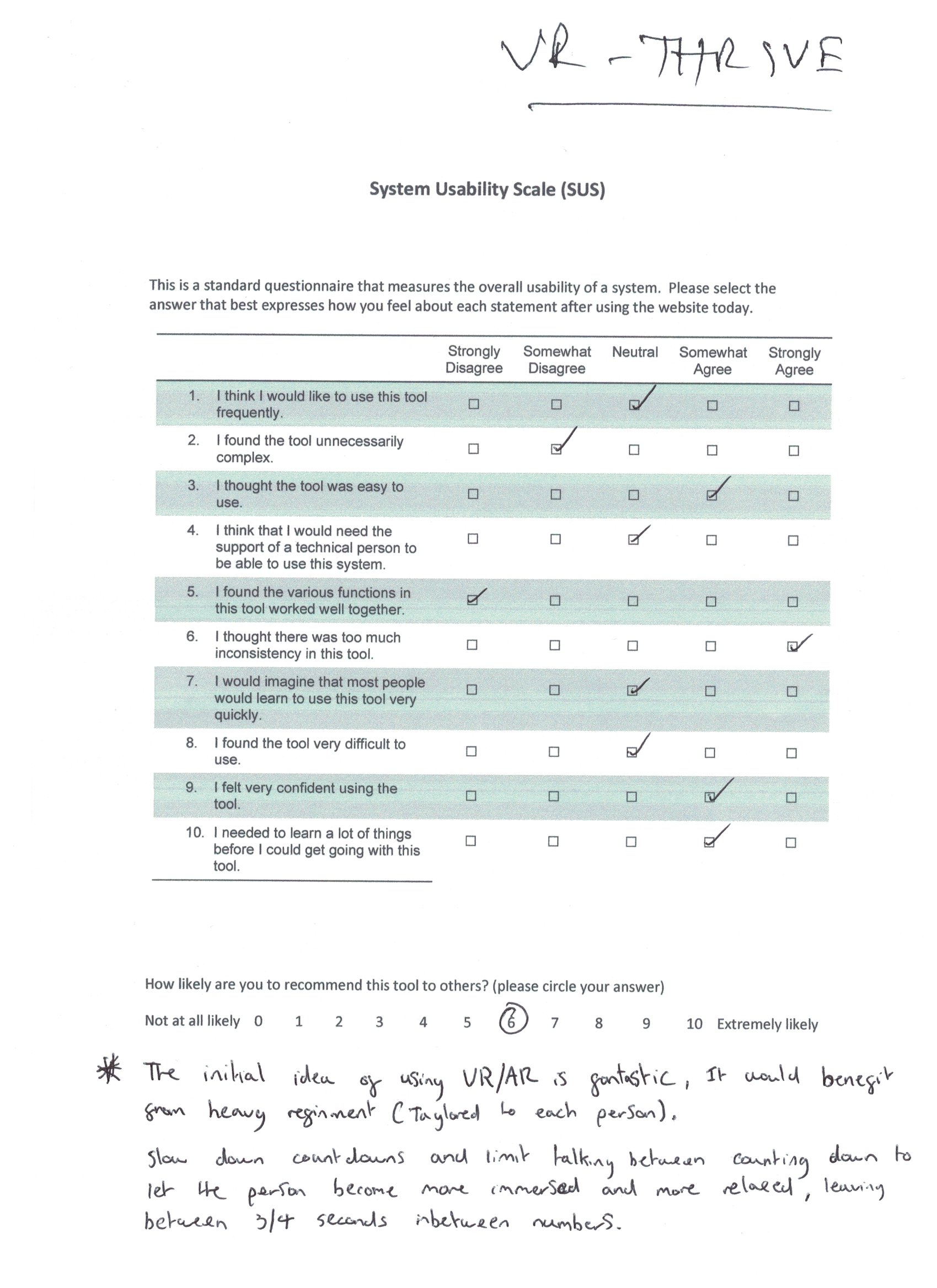

Supplement: Multimedia Appendix 8 [file xr-v2-e68580-s008.zip › Relaxation/Scan_3.png]

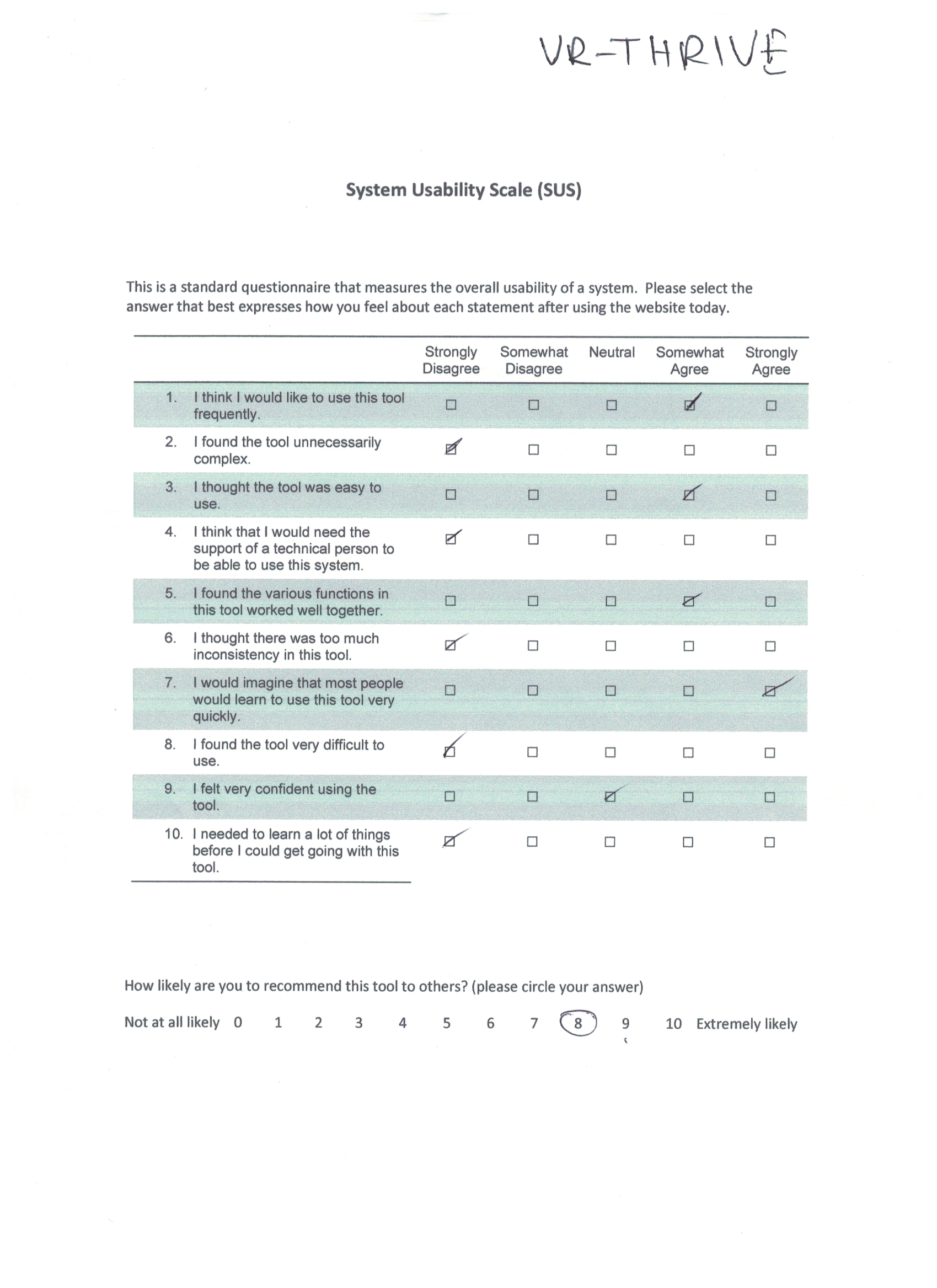

Supplement: Multimedia Appendix 8 [file xr-v2-e68580-s008.zip › Relaxation/Scan_4.png]

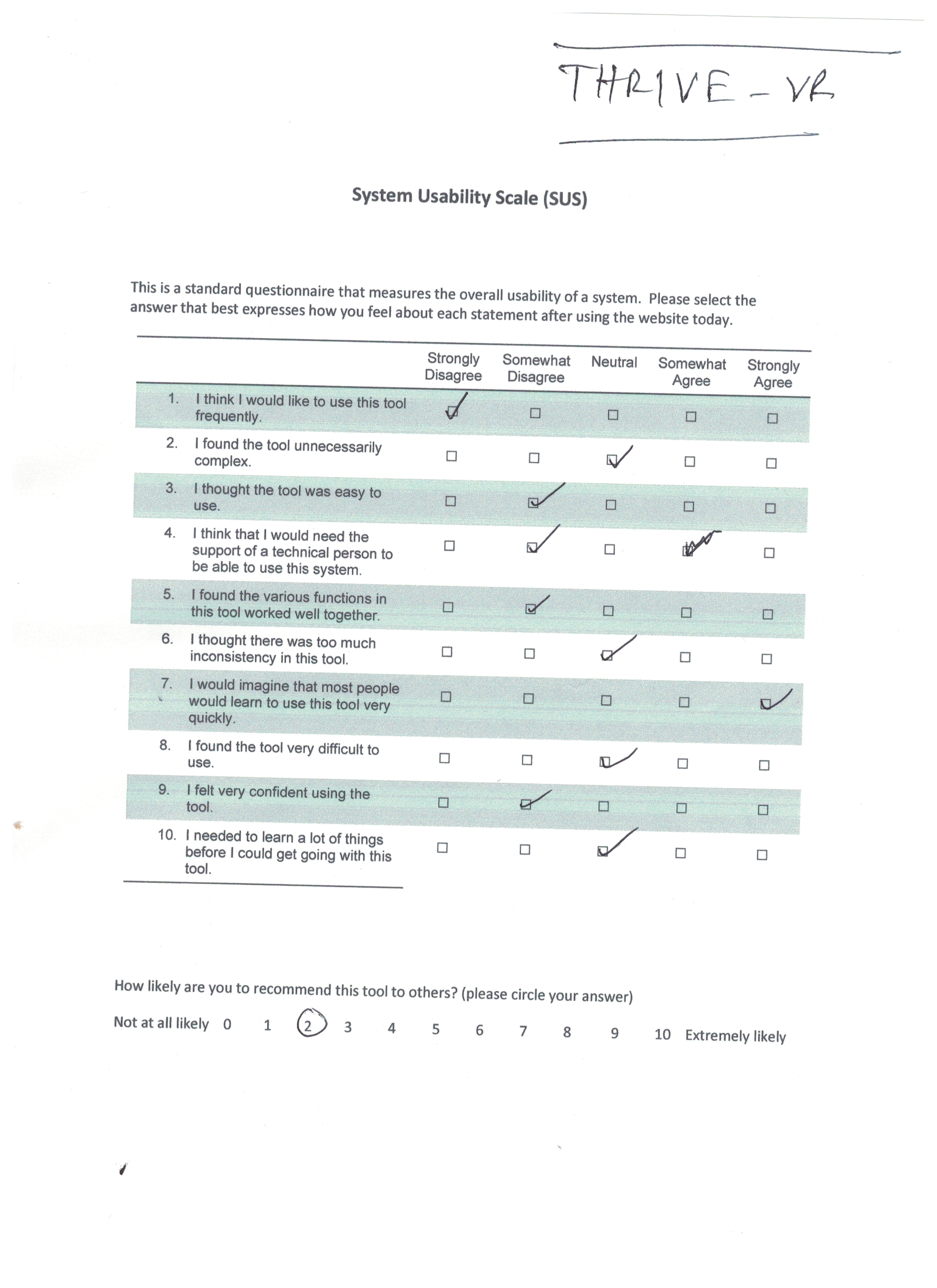

Supplement: Multimedia Appendix 8 [file xr-v2-e68580-s008.zip › Relaxation/Scan_5.png]
